# Learning Speech Production and Perception through Sensorimotor Interactions

**DOI:** 10.1093/texcom/tgaa091

**Published:** 2020-11-27

**Authors:** Shihab Shamma, Prachi Patel, Shoutik Mukherjee, Guilhem Marion, Bahar Khalighinejad, Cong Han, Jose Herrero, Stephan Bickel, Ashesh Mehta, Nima Mesgarani

**Affiliations:** 1 Department of Electrical and Computer Engineering, Institute for Systems Research, University of Maryland, College Park, MD 20742, USA; 2 Laboratoire des Systèmes Perceptifs, Department des Etudes Cognitive, École Normale Supérieure, PSL University, 75005 Paris, France; 3 Department of Electrical Engineering, Columbia University, New York, NY 10027, USA; 4 Mortimer B Zuckerman Mind Brain Behavior Institute, Columbia University, New York, NY, USA; 5 Neurosurgery, Hofstra Northwell School of Medicine, Manhasset, NY, USA; 6 The Feinstein Institutes for Medical Research, Manhasset, NY 11030, USA

**Keywords:** auditory cortex, human ECoG, mirror network, sensorimotor interactions, speech perception, speech production, vocal articulation

## Abstract

Action and perception are closely linked in many behaviors necessitating a close coordination between sensory and motor neural processes so as to achieve a well-integrated smoothly evolving task performance. To investigate the detailed nature of these sensorimotor interactions, and their role in learning and executing the skilled motor task of speaking, we analyzed ECoG recordings of responses in the high-γ band (70–150 Hz) in human subjects while they listened to, spoke, or silently articulated speech. We found elaborate spectrotemporally modulated neural activity projecting in both “forward” (motor-to-sensory) and “inverse” directions between the higher-auditory and motor cortical regions engaged during speaking. Furthermore, mathematical simulations demonstrate a key role for the forward projection in “learning” to control the vocal tract, beyond its commonly postulated predictive role during execution. These results therefore offer a broader view of the functional role of the ubiquitous forward projection as an important ingredient in learning, rather than just control, of skilled sensorimotor tasks.

## Introduction

Sensorimotor interactions have long been postulated as a fundamental ingredient of performance of complex tasks engaging a perceptual system (visual, auditory, or somatosensory) and a concomitant suite of motor actions (reaching, speaking, and lifting) (Wolpert and Ghahramani 2000; Keller et al. 2012). The conceptual motivations are anchored in control theory where rapid complex actions can benefit from fast sensory feedback to inform the controllers of the accuracy of the ongoing performance so as to maintain or correct its course (Conant and Ashby 1970; Wolpert et al. 1995). The same rationale and motivations also apply in purely sensory contexts where the balance between bottom-up stimulus representations and its top-down predictions are postulated to play a key role in stimulus perception (Keller and Mrsic-Flogel 2018).

Feedback may take the form of deviations (errors) between the sensory consequences of an ideal target performance and its “prediction,” computed by extrapolating a “forward” model of the motor-plant. This is how accurate arm reaching is informed by visual and proprioceptive cues (Jackson and Husain 1997) and how the vocal tract exhibits smooth delivery and executes rapid corrections of speech from auditory feedback (Hickok 2012; Houde and Chang 2015; Wirthlin et al. 2019). This predictive function of sensorimotor interactions has even been postulated to apply in reverse, to explain how robust sensory perception can arise from observing motor action, for example, the role of lip-reading in speech comprehension, or in the Motor Theory of Speech where acoustic features of speech are presumed to be transformed and encoded as articulatory commands (Liberman et al. 1967; Massaro and Chen 2008; Lotto et al. 2009). Finally, these bidirectional sensorimotor interactions achieve their full generalization in the findings of the mirror-neuron responses (Perry et al. 2018), which have claimed a causal role not only in all sensorimotor systems but also in accounts of social function and emotional relations (Iacoboni 2009). Predictably, these claims have provoked numerous detractions and debates that have served to enrich and deepen the understanding of these phenomena.

**Figure 1:**
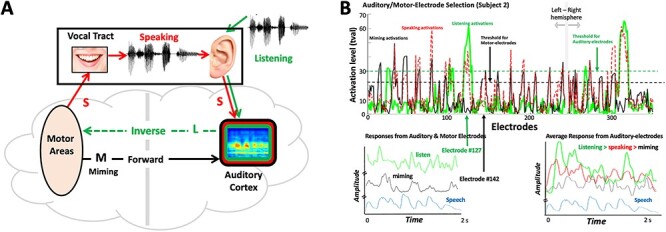
Experimental Paradigms. (*A*) Schematic depicts the four types of recordings from all electrodes which are expected in each subject: **Miming** (**M**) responses are when a subject articulates the speech without any sound; **Listening** (**L**) responses are from the subject listening passively to the speech; **Speaking** (**S**) signals are recorded while subject articulates audibly the speech; **Noise** (**N**) are recordings of the background noise on the electrodes in silence. The schematic illustrates the postulated *forward* and *inverse* projections between the auditory and motor areas. (*B*) **Electrodes selection:** (Upper panel) Recordings were usually made with numerous electrodes, for example, 353 in this subject (240 in right hemisphere). Only a few electrode responses were selected for analysis, according to the illustrated criteria. Specifically, auditory electrodes are those that exceed specific activation levels during listening (green trace), for example, tval-threshold = 30. Motor electrodes are those that exceed a specific threshold of activation during miming (black trace), for example, tval threshold = 20. Speaking usually activates both auditory and motor electrodes (dashed red trace), and even sometimes additional electrodes that are not in either set. However, these are not used in this analysis. (Lower left panel) Responses from an auditory electrode (#127) during listening (green trace), and a motor electrode (#142) during miming (black trace) to the same speech stimulus (signal envelope in blue trace). (Lower right panel) Average responses from all auditory electrodes during listening (green), speaking (red), and miming (black) to the same speech sentence (signal envelope in blue trace). All responses are plotted on the same amplitude scale and baseline. Therefore, they are largest during listening, smaller during speaking, and smallest during miming.

In order to characterize sensorimotor interactions in the human cortical speech system, we recorded and analyzed the sensorimotor neural interactions with ECoG in humans while they spoke, listened, or simulated speaking by moving their vocal tract without producing sound. The goal was to characterize more accurately the nature of the spectral or temporal representation of the auditory and motor cortical responses. We also used these responses to re-examine the basic computational architecture of the sensorimotor interactions with the aim of clarifying their functional role in action and perception. Figure 1*A* illustrates the basic reciprocal sensorimotor projections as would typically be involved in speech production (Poeppel 2014; Houde and Chang 2015). Specifically, during speaking, motor areas control vocal-tract movements that generate a speech signal. It has also been proposed that certain motor cortical areas send a parallel internal neural copy of the speech signal to the auditory cortex—the forward prediction signal, where it is compared with the responses induced by the incoming speech (Hickok and Poeppel 2007). During listening to speech, an “inverse” mapping from the auditory to the motor areas would create a motor representation of the acoustic signals (Wilson et al. 2004).

Because of this bidirectional flow of interactions between the auditory and motor responsive regions (**L** and **M** in Fig. 1*A*), we shall refer to this phenomenological network as the “Mirror Network” (or MirrorNet). In the context of this framework, we explain how ECoG recordings directly reflected the spectrotemporal nature of the MirrorNet projections: the forward motor influences into the auditory cortex during silent speaking (or miming), the inverse auditory influences into the motor areas during listening, and finally the bidirectional influences during speech production. Two previous studies (Cogan et al. 2014; Martin et al. 2018) had adopted experimental paradigms analogous to ours. However, the goals, analyses, and conclusions differ fundamentally from those of this study, although they are mutually consistent as we shall elaborate later. Finally, it should be emphasized that we use the terms “motor” and “auditory” here to refer to the dominant sources of the forward and inverse projections that we seek to contrast; a myriad of other influences likely contribute to or modulate these auditory and motor signals such as those due to imagination, expectations, linguistic processes for lexical access and sentence formation, and other cognitive functions that cannot be fully controlled for or eliminated (Skipper et al. 2017), but nevertheless can still be contrasted to learn from their differences.

The findings from our experiments confirm the basic structure of the auditory–motor mirror network (Fig. 1*A*) and reveal that the responses of the forward and inverse projections are spectrotemporally rich enough to allow for accurate representations of speech. The results also suggest that a key function of the sensorimotor interactions is to enable the brain to learn how to use the vocal tract for speech production, rather than simply to control its performance during speaking. In support of this idea, we developed a computational instantiation of this basic network and used it to train a speech synthesizer to produce speech from mere exposure to a corpus of speech data, thus demonstrating how complex actions like speaking or playing a piano can be learned through auditory feedback and motor feedforward signals between the two cortical regions.

## STAR Methods

Contact for reagent and resource sharing of further information and requests for resources and reagents should be directed to and will be fulfilled by the Lead Contact, Prof. Nima Mesgarani (nima@ee.columbia.edu).

### Human Subjects

Four subjects (aged: 20, 22, 50, 51) participated in this study while undergoing clinical treatment for epilepsy. All subjects gave their written informed consent to participate in research. All subjects were located at North Shore University Hospital (NSUH). Research protocols were all approved and monitored by the institutional review board at the Feinstein Institute for Medical Research and at Columbia University. Informed written consent to participate in research studies was obtained from each subject before implantation of electrodes. All subjects had depth electrodes implanted, with varying amounts of coverage over the left and right auditory and motor cortices for each subject.

## Method Details

### Stimulus

Natural American English sentences were presented varying in duration from 1 to 2 s from the TIMIT database. A computer screen was placed in front of the subjects to cue them about the task. Sentences were presented one at a time using a single Bose SoundLink Mini 2 speaker in front of the subject. The cue on the screen would read (in the following order): “Listen” (**L**): indicating the subject that they have to listen to the sentence presented; “Loud Articulation” or speaking (**S**): indicating to the subject that they have to repeat the sentence they just heard loudly; “Silent Articulation” or miming (**M**): instructing the subject to repeat the sentence silently without any sound; “Listen”: instructing the subject to listen to the same sentence, but now played in their own voice; and “Silent Articulation”: the subject repeats the sentence once again silently. Only 3 subjects had the screen in front of them for the cue. In one subject (#4), we did not use the screen to cue them and consequently collected limited data (one presentation only) in the various **L, S,** and **M** conditions. To segment the brain responses for each of these task conditions, we recorded a video and audio of the subject performing the task which was synced to the recording of their neural data.

Subsequent data analysis relied on segmenting all the recorded stimuli and video into sentence-long segments, and aligning all **L, M,** and **S** responses to the speech stimuli. In all scenarios, the acoustic spectrograms (for **L** and **S** conditions) were used as the reference to align the responses. In the **M** condition, there were no acoustic signals, and so the videos of the subjects articulating silently the sentences were used. The frame rate of the video was 10 frames/s, and the beginning of a sentence was estimated based on a careful visual inspection of when the subject began to articulate. Extra care was taken to minimize systematic temporal misalignments between the **M** responses and their corresponding speech spectrograms. One independent check of such misalignments is seen in the **M**-STRF’s measured on motor and auditory electrodes. Those responses were aligned exactly the same way relative to the speech spectrograms, yet they exhibited a significantly different latency relative to the onset of the stimuli, which we conclude reflect a functionally meaningful temporal shift in the responses relative to the onset of the articulations.

Noise samples were collected on all electrodes during 10 s of silence at the start of the recording sessions. To extend the signal to match the lengths of the **L**, **S**, and **M** recordings, we modeled the noise samples of each electrode as the 10th order AR processes. The AR coefficients are estimated by the OLS method and used to generate new noise signals of the appropriate duration. The mean and variance of the generated noise signals are matched to those of the 10 s of recorded spontaneous activity.

### Data Preprocessing and Hardware

Electrocorticography signals with sampling rate of 3000 Hz were recorded with a multichannel amplifier connected to a digital signal processor TDT (Tucker-Davis Technologies). All data was montaged again to common average reference (Crone et al. 2001). Neural responses were first filtered using the Hilbert transform to extract the high-gamma band (70–150 Hz) for analysis (Edwards et al. 2009) and were then down-sampled to 100 Hz for further analysis.

### Auditory and Motor-Electrode Selection

Electrodes were designated as auditory electrodes or motor electrodes depending on what drove their responses. Locations of some of these electrodes in 3 subjects are shown in Supplementary Figure 5.3**.** Auditory electrodes were those that responded to speech during passive listening, but not during silent miming. These sites were determined by calculating the maximum *t*-value of each electrode’s response between silence and speech. Electrodes with a maximum *t*-value greater than a threshold value (*t*-values>30 (*P* < 0.001)) were selected (Fig. 1*B*), resulting in 9, 15, 17, and 13 electrodes from subjects 1–4, respectively, for a total of 54 electrodes used in most further analyses. Motor electrodes were similarly selected by their activity during silent miming of the speech sentences [*t*-values>20 (*P* < 0.001), Fig. 1*B*], resulting in 21 electrodes from each of subjects 1–3 for a total of 63 electrodes. Subject #4 had no responsive motor coverage. For all subjects, no electrodes responded significantly enough in both listening and miming conditions, and hence, the two sets of electrodes were mutually exclusive.

### Spectrotemporal Receptive Fields and Stimulus Reconstruction

We calculated the spectrotemporal receptive fields (STRF) of each electrode using a normalized reverse correlation algorithm. Regularization and cross-validation techniques were used to prevent overfitting of the STRF (David et al. 2007). STRFs were calculated using the same input speech spectrograms, and responses during the three different conditions: Listening (**L**-STRF), Speaking (**S**-STRF), and Miming (**M**-STRF). The different STRFs were examined either individually (per electrode) or averaged over all auditory and motor electrodes. To confirm the meaningful nature of these STRF measurements, we confirmed in all cases that the STRFs lose their structured look when we shuffled the labels of the sentences relative to the responses. Furthermore, we also confirmed that excising up to 50 ms of the onset responses and their corresponding speech stimuli did not affect the shapes of the STRFs, indicating that they reflected primarily the correlations between the ongoing phase-locked responses and their corresponding speech stimuli, and not just the onsets.

We also estimated using the same methodology the relationship between auditory- and motor-electrode activities by treating them as inputs and outputs and computing the “filter” that transforms one to the other using the same reverse-correlation algorithm.

Stimulus reconstruction decoders were calculated (Mesgarani et al. 2009; Pasley et al. 2012) using custom code to implement ridge regression. K-fold cross-validation was used to select a ridge parameter that would optimally predict neural data in the case of an STRF or optimally reconstruct spectrograms in the case of stimulus reconstruction.

### Generation of Brain Figures

This study was not specifically designed to localize the sensorimotor interactions, but rather to explore the dynamics of the sensorimotor interactions. Therefore, there was no attempt to optimize the distribution of the electrode recordings across the various brain regions, and consequently, several regions were highly under-represented, and a few electrodes were difficult to localize because of their placement relative to skull screws and other technical reasons. Nevertheless, we managed to map many of the electrodes in each subject using co-registration by iELVis (Groppe et al. 2011, 2017) followed by their identification on the post-implantation CT scan using BioImage Suite (Papademetris et al. 2006). Anatomical locations of these electrodes were obtained using Freesurfer’s automated cortical parcellation (Dykstra et al. 2012) by destrieux brain atlas (Destrieux et al. 2010). These labels were closely inspected by neurosurgeon using subject’s co-registered post-implant MRI. The electrodes were plotted on the average brain template ICBM152 (Fonov et al. 2011) using Brainstorm (Tadel et al. 2011). We were able to localize accurately most auditory electrodes. However, in two subjects (1,2), it proved difficult to be certain of the locations of a subset of the motor electrodes. All those were labeled *UC* (uncertain). Other locations are labeled as follows: Superior-temporal gyrus and sulcus (STG, STS); inferior and middle temporal gyrus (ITG, MTG); Heschel gyrus (HG); planum temporalis (PT); precentral and post-central gyrus (PG, PCG); insula (INS); anterior lateral fissure; caudal middle frontal; inferior opercular sulcus; superior frontal gyrus; and hippocampus. Supplementary Figure 5.3 gives an overview of the electrode placements in three subjects. Orange and blue electrodes refer to auditory and motor electrodes, respectively. Darker shades of these colors refer to electrodes that were relatively strongly interacting electrodes.

### Electrode Receptive Fields between Auditory and Motor Electrodes

Electrode receptive fields between the two sets of auditory and motor electrodes in each subject were calculated in the same manner as the STRFs (i.e., k-fold ridge regression). Time lags from −100 to +300 ms were used in the analysis. The two sets of electrodes were commonly quite far apart, and hence, their noise correlations were relatively weak compared with the evoked-response correlations. We further used the prediction quality of these auditory or motor receptive fields to weight the display of each electrode’s mapping. Thus, the more predictable electrodes have more strongly modulated receptive fields and hence more vibrant colors.

## Quantification and Statistical Analyses

### Response Correlations across Conditions and Electrodes

Correlation coefficients **cc**^***ij***^ were computed to measure the match between the responses across electrodes or conditions, as well as between reconstructed and original spectrograms. Unless explicitly stated, all responses were normalized to have a zero-mean and unit variance. Comparisons between the spectrograms and reconstructed spectrograms were often done on a per-frequency-channel case, with the matches then all averaged at the end. We also computed the **cc**^***ij***^ on the full normalized spectrograms, with very similar results.

### Ranking Method for Sentence Recognition

To assess whether responses to the 60 sentences reflected specifically the spectrotemporal structure of the stimuli that evoked them, we computed the correlation-coefficients **cc**^***ij***^ in the following two sets of tests:

1. **cc** ^***ij***^ **= < M**^***i***^**, L**^***j***^**>,** where **M** is the raw response to the *i*th sentence and **L** is the response to the *j*th sentence. If the responses are accurate enough, then this **cc**^***ij***^ should be largest when both the **L** and **M** responses are to the same sentence, that is, when ***i* = *j*** for all sentences.
2. **cc** ^***ij***^ **= < M**^***i***^**, L**^***j***^**>,** where **M**^***i***^ and **L**^***j***^ are the reconstructed stimulus spectrograms from all **M** and **L** responses on all electrodes, to the ***i*th** and ***j*th** sentences, respectively. Again, if the reconstructions reflect accurate spectrotemporal responses, then the **cc**^***ij***^ should be largest when the two reconstructions are of the same sentence, that is, ***i* = *j*** for all sentences.

We computed the **cc**^***ij***^ values across all sentences and then ranked these values for each ***i*th** sentence against all other 60 sentences. We normalized the ranks between 0 and 1, where 1 refers to the highest and 0 is the lowest rank (among all 60 different sentences). We then combined the data from all matches and computed the average rank of the **cc**^***ii***^ for all 60 sentences and compared the average to a random shuffling of all sentence labels. The more reflective the responses are of the sentences that evoke them, the better is the rank of the **cc**^***ij***^.

### Implementation and Training of the MirrorNet

The MirrorNet is a model for learning to control the vocal tract based on an auto-encoder neural network architecture. The structure of this network is shown in Figure 6, which is functionally equivalent to the projections and measurements depicted in Figure 1*A*, and is arrived at as detailed in the beginning of the last section in **Results** entitled “Sensorimotor interactions and learning in the Mirror Network.” The goal of the MirrorNet is to demonstrate the potential function of the sensorimotor projections in learning how to control the vocal tract by generating the appropriate motor commands corresponding to any intended speech signal. This entails learning the two neural projections investigated in the analysis of this study (Fig. 6*A*): an inverse mapping from auditory representation to motor parameters (Encoder) and a forward mapping from the motor parameters back to the auditory representation (Decoder). As a model of the vocal tract, we used the WORLD synthesizer (Morise et al. 2016), a simple and widely used speech synthesizer. A python wrapper of the original code was used in this study (https://github.com/JeremyCCHsu/Python-Wrapper-for-World-Vocoder). The MirrorNet model also consisted of multilayer convolutional neural networks as the Encoder and the Decoder.

The WORLD synthesizer takes in as input a set of parameters at each time instant representing the spectral envelope of a speech (SP), the pitch (F0), and the voicing/no-voicing indicator (AP) and generates a time-waveform with a spectrogram of these features. The overall goal is for the Encoder to invert this process and produce the parameters (SP, F0, and AP) from the waveform and for the Decoder and synthesizer in parallel to reproduce the same waveforms. All waveforms are actually converted to their corresponding auditory spectrograms, and all the errors used in the learning process are measured in the spectrogram domain. The dimensions of F0, SP, and AP were 1, 513, and 513, respectively, for each 5-ms segment of speech, and the generated speech from the synthesizer was sampled at 16 kHz. Thus, for a 2-s waveform (1*32 000), F0, SP, and AP are 1*400, 513*400, and 513*400 long, respectively. The neural networks were based on a multilayered Temporal Convolutional Network (Lea et al. 2017) using *RELU* as activation functions. This network implementation is illustrated in Figure 6*C*.

The Encoder and Decoder networks are trained to map the input spectrogram (S) to the parameters and back again to a reconstruction of the spectrogram S′, which in parallel with the synthesizer output S″ (Fig. 6*A*). The training objective is to minimize both the error **e**_**c**_ between (S**′**, S) and the error **e**_**d**_ between (S**′**, S**′′**). We thus consider the MirrorNet very similar to a classic “autoencoder network,” but with a constraint that the output of the synthesizer S**′′(**F0, SP, AP**)** is mapped to →S**′** by minimizing **e**_**d**_ and simultaneously minimizing **e**_**c**_ to map S**′**→S. Consequently, the adjustments of the Encoder and Decoder networks are carried out simultaneously through a backpropagation of the errors as explained in the text. The speech database was obtained from the CSTR VCTK Corpus which contains about 12 000 sentences. Each waveform is resampled at 16 kHz, trimmed to 2 s, and normalized to unit power https://doi.org/10.7488/ds/1994.

Training was performed on 194 batches, each of size 64 sentences using the Optimizer Adam. The initial learning rate is 10^−3^ and took about 60 min to train 1 epoch. *Training strategy:* The key procedure that led to a successful training of the MirrorNet is to perform the training epochs alternately minimizing **e**_**d**_ and **e**_**c**_. There were 2 phases to the training. ***Phase 1:*** The initialization of the training proceeded by using random assignments of the “hidden parameters” **F0**, **SP**, and **AP**, which are used to generate through the synthesizer an initial random spectrogram and then to minimize the error **e**_**d**_ to have the Decoder converge toward the synthesizer. At the same time, the random spectrogram is used to initialize the Encoder to map it to the random **F0**, **SP**, **and AP**. This initialization proceeds with many random **F0**, **SP**, and **AP** equivalent to about 20 min of speech, and the error **e**_**d**_ decreased considerably. ***Phase 2**:* Using 20–40 min of natural speech material, the initialized network continued to be trained with alternating epochs and decreasing **e**_**d**_ and **e**_**c**_ for at least another 20 min. The results of the training illustrated in Figure 6*D* used unseen material network after training was stopped. Clearly, the errors continued to decrease, and higher fidelity is assumed to be possible if training continues with more speech.

### Data and Code Availability

There are restrictions to the availability of dataset due to the protection of human subjects who participated in this study. The data that support the findings of this study are available upon request from the corresponding author [NM].

## Results

Recordings were obtained with ECoG electrodes implanted in 4 patients during surgery to relieve epileptic seizures (see Supplementary Fig. 5.3 for some electrode locations in 3 of the patients and Methods). Neural responses were recorded under four different scenarios as illustrated in Figure 1*A*: **1) Listening (L)**, where subjects listened to a sequence of 60 sentences selected from a speech corpus (TIMIT, Zue et al. 1990); after each sentence, subjects **2) Spoke (S)** audibly repeating the sentence they just heard. They then **3) Mimed (M**) the same sentence *without* producing any sound, and finally, a sample of **4) Noise (N)** was recorded while the subjects remained silent. For 3 subjects, the **L** and **M** scenarios were repeated using the subjects’ own spoken utterances, and these are the primary sources of the results of the analyses described below. The contrast between the **M** scenario and the others was utilized in a similar fashion in Cogan et al. (2014), and more recently by Martin et al. (2018) for playing a musical instrument with and without sound, and hence some of our analyses and interpretations echo these studies.

Surface and implanted electrodes were placed on each subject, distributed over a wide cortical area with coverage in HG, STG, several Motor areas, and many other regions. For all our analyses, the ECoG responses refers to the envelope of the γ-band activations (70–110 Hz) extracted by filtering the raw electrode signals; these responses are thought to approximately reflect aggregate neural activity in a local region (Ray et al. 2008; Steinschneider et al. 2008; see Methods for details). Two sets of measurements were used from electrodes that were selected based on the strength of their responses in the **L** and **M** conditions as illustrated in Figure 1*B*: Auditory electrodes are those that respond strongly (activation criteria: *t*-values of the *t*-test 30) when the subject listens passively to speech. Motor electrodes are those that respond strongly (activation criteria: *t*-values 20) when the subject mimes the speech (**M**) without any sound. The number of such selected electrodes varied across patients from 9 to 30 electrodes for each set. We have observed that electrodes strongly activated by miming exhibited *t*-values <10 during listening, and vice versa. Hence, classifying electrodes as either auditory or motor according to the threshold criteria mentioned above resulted in electrodes that were either classified as auditory or motor, but never both. Figure 1*B* illustrates the typical activation patterns in subject 2 electrodes, threshold levels for selecting the electrodes from two hemispheres, and the time waveforms in response to listening to speech on three arbitrarily selected auditory electrodes. We emphasize again that while threshold levels in Figure 1*B* are somewhat arbitrary (e.g., *t*-values > 20), the results reported here remain unaltered by the choice of slightly different thresholds (and hence electrodes) as long as the two sets of selected electrodes remain largely mutually exclusive in the way described above. Finally, we stress that the designation of the electrodes as auditory or motor in this study is a functional definition based on their predominant responsiveness to auditory and motor stimuli and not on their anatomical locations.

We begin by analyzing separately the global response patterns accumulated from all auditory and motor electrodes. We focus first on the encoding of auditory responses evoked by silent motor activity (during **M**), that is, the forward projections in Figure 1*A*. Then, we examine the complementary motor responses induced during passive listening to sound (during **L**), that is, the inverse projections. Our aim here will be to characterize the spectral and temporal nature of the activity conveyed by both these projections. Subsequently, we shall dissect in more detail the contributions of the individual electrodes to the various overall global interactions and, where possible, identify their anatomical locations over the auditory and motor responsive areas. Based on these findings, we shall then explore the functional significance of these projections in the context of speech production and perception via mathematical modeling and simulation of the Mirror Network.

### Spectrotemporal Specificity of Auditory-Electrode Responses Induced by Motor Activity

We begin by exploring the responses due to the *forward* projections postulated in Figure 1*A*, namely, the responses in the auditory regions (electrodes) presumably induced primarily by the motor activity of silent articulation, or miming (**M**). We sought to determine the nature of these responses on the auditory electrodes by comparing them to the neural activity during other scenarios: **L**, **S**, and **N**. Data were accumulated from all 4 subjects and electrodes to enhance the statistical significance of the findings, although results from individual subject were consistent with the overall findings (see Supplementary Figures). Four complementary analyses were conducted to test if the vocal-tract motion evokes auditory-like responses in auditory regions that are significant and sufficiently detailed to allow a reconstruction of the speech stimuli.

### Response Correlations across Different Conditions

Auditory electrodes were (as expected by design) most responsive during listening (**L)** and were relatively suppressed during speaking (**S)** down on average to 75% of **L**  *r.m.s.* response power (Fig. 1*B*; lower right panel). This finding has been reported previously in numerous recordings and imaging studies (Paus et al. 1996; Curio et al. 2000; Agnew et al. 2013; Houde et al. 2002; Eliades and Wang 2003; Heinks-Maldonado et al. 2005). By comparison, **M** responses were weak at 55% of **L** on average (Fig. 1*B*; lower right panel), but still higher than the average level **N** at 35% of **L.**

**Figure 2:**
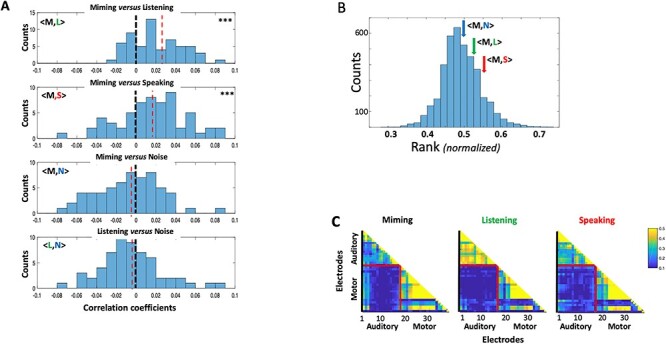
Correlations of auditory-electrode responses in different recording conditions. (*A*). Distributions of the pairwise correlation-coefficients between **M** versus **L** (<**M,L**>) and **M** versus **S** (<**M,S**>) responses to the same speech stimuli, and also **M** and **L** versus noise **N** activity on the same auditory electrodes (<**M,N**>) and (<**L,N**>). Data are aggregated from all subjects and electrodes. (Top two panels) **M** responses are positively correlated with **L** and **S** responses, and hence the distributions are positively shifted relative to the lower two panels (*P* < 0.001; 2-sample *t*-test). (Lower two panels) Neither **M** nor **L** are significantly correlated with **N**. (*B*) The average rank of correlation coefficients between responses during **M** and other conditions (**L, S,** and **N**). The distribution of ranked correlations from randomly ordered responses to different sentences is shown together with the arrows signifying the average <**M,L**> and <**M,S**> on the same sentences. The latter were modestly different from the mean (0.63**σ** and 1.13**σ**, respectively) compared with the average of noise response correlations <**M,N**> (0.15**σ**). (*C*). The correlation coefficients between all pairs of auditory and motor electrodes in subject 3, all measured using the responses within each condition separately. On average, the correlations between the auditory and the motor electrode sets are quite weak compared with within each electrode set. This suggests that the responses across the two electrode regions are of a different nature.

A key question we sought to answer concerned the nature of the **M** responses relative to **L** and **S** and specifically whether the temporal response modulations reflect the spectrotemporal structure of the acoustic speech stimuli. One indicator of such a relationship is if the responses to the **M** had significant “meaningful” correlation with both **L** and **S** responses measured on the same electrode. Figure 2*A* illustrates the distribution of such pairwise correlation coefficients <**M,L>** and <**M,S>** accumulated from all auditory electrodes in 4 subjects. In both cases (top 2 panels), there were significant positive correlations (***P*** < 0.001, 2-sample *t*-test) confirming a resemblance between the temporal structure of the responses among the 3 response conditions. This conclusion is further supported by the absence of such a positive bias in the correlation coefficients between **M** or **L** and the noise **N** (histograms of **<M,N>** and **<L,N>** in the lower 2 panels of Fig. 2*A*). Therefore, we conclude from these data that despite the absence of sound, vocal-tract motion during **M** evokes responses that resemble auditory responses which results in significant correlations with the responses during **L** and **S** conditions, but not in the noise **N**.

It should be noted that these recordings are noisy and the responses in all conditions are small. This is true even for the nominally large response conditions of **L** and **S** which typically yield mutual correlation coefficients of about 0.1. Furthermore, the wide scatter in the correlation distributions indicates that response patterns in the different conditions vary considerably relative to each other across the auditory electrodes.

To assess more closely the fidelity of the **M** responses relative to those of **L** and **S**, we tested whether the **M** responses preserved enough spectrotemporal details to discriminate among the different speech sentences. To do so, we segmented and labeled all responses to the 60 sentences in the different conditions and then computed correlations between the **M** versus **L** responses to the same and across all sentences. For high-fidelity responses, that is, temporally modulated and spectrally localized, it is expected that the correlation between **M** and **L** responses to the same *i*th sentence (*r_i,j_* = <**M**_**i**_,**L**_**i**_**>**) is ranked higher than the correlations between responses belonging to different sentences (e.g., *r_i,j_* = <**M**_**i**_,**L**_**j**_>). Therefore, by rank ordering all the correlations (with the lowest to highest normalized to between 0 and 1), we can estimate the average rank attained by the same sentence correlations from all sentences and compare it to the rank distribution for randomly labeled sentences. Figure 2*B* displays the average ranks for correlations between **M** and recordings from each of the other three conditions (**L**, **S**, and **N**) and how these compare to the random distribution of ranks. Thus, both <**M**,**L**> and <**M**,**S>** correlations accumulated from all auditory electrodes and subjects are modestly above the average and higher than <**M**,**N>** correlations (quantified in the figure legend), suggesting that **M** has meaningful response correlations with those of **L** and **S**. These results are consistent with distribution patterns of the three correlation coefficients noted in Figure 2*A*.

Finally, we computed the correlations between simultaneously recorded responses on the auditory versus the motor electrodes during **L**, **M**, and **S** conditions. The goal was to determine if the responses shared a similar detailed temporal structure. Figure 2*C* illustrates the results in subject 3, which demonstrate that under **M**, **L**, or **S** conditions, auditory-electrode responses were only weakly correlated with motor-electrode responses, typically less than 10% of the average correlations seen within the auditory or within motor-electrodes. This finding was true of all subjects (Supplementary Fig. 2), suggesting that the responses in the auditory and motor electrodes are different in nature, consistent with previous measurements (Arsenault and Buchsbaum 2016; Cheung et al. 2016), and as we shall elaborate later.

### Spectrogram Reconstructions from Auditory-Electrode Responses

A different approach to dissecting the details of cortical responses is to reconstruct the stimulus spectrograms that evoked them (Mesgarani et al. 2009). The advantage of this method is that it integrates and maps all electrode responses from all subjects to the same stimulus spectrogram space, where they are easier to visualize, interpret, and compare to the original stimulus spectrograms. Specifically, the more spectrotemporally accurate the responses are, the better are the reconstructions of the stimuli.

**Figure 3:**
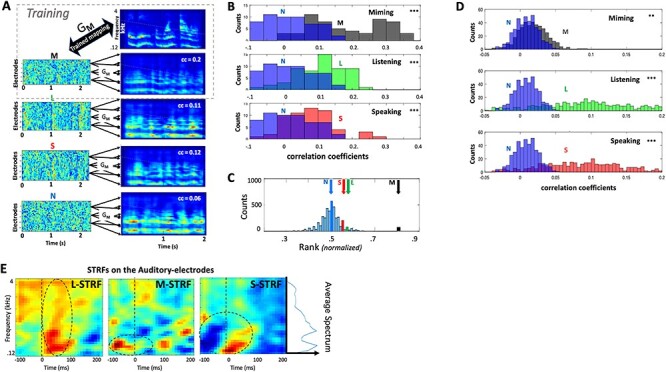
Analyses of responses on auditory electrodes. (*A*). Illustration of stimulus reconstruction procedures. **M** responses from 28 auditory electrodes in subject 2 were trained to reconstruct (through filter **G**_**M**_) the spectrograms of all speech stimuli that evoked them. **L** and **S** responses share sufficient details with **M** responses, such that applying the **G**_**M**_ to them also reconstructs similar spectrograms, as exemplified in the panels. The mapping fails to reconstruct a good spectrogram from the noise **N**. (*B*). Spectrogram reconstructions from **M**, **L**, and **S** responses are compared with all stimulus spectrograms, and accumulated from all frequencies, electrodes, and subjects. The correlation coefficients are depicted as histograms (**M**—black; **L**—green, **S**—red) each against the histogram of the **N** reconstructions (blue). The **M**, **L**, and **S** distributions are significantly shifted upwards relative to the **N** (*P* < 0.001; 2-sample *t*-test), indicating better matches to the original spectrograms, and hence the presence of spectrotemporal structure related to the stimuli (see text for more tests and details). (*C*) The average rank order of the correlation between a sentence and its corresponding reconstruction, compared with all other sentence comparisons. Reconstructions from **M**, **L**, and **S** response conditions are sufficiently accurate to allow reasonable recognition of the corresponding stimuli with above chance accuracy (6.75**σ**, 1.55**σ**, and 1.26**σ**), where **σ** = **0.0**027. Reconstructions from **N** responses perform at chance level (0.02**σ**). (*D*). Distribution of the correlation coefficients between original and reconstructed spectrograms based on training G_**M**_, G_**L**_, G_**S**_, and G_**N**_ filters on selected response segments, and cross-validated with predictions from unseen segments. Distribution of the correlation-coefficients indicate that **N** (blue) responses produce worse predictions than **M** (black), **L** (green), and **S** (red) conditions. (*E*). Average STRFs from all auditory electrodes in subjects 1, 2, and 3. (Left-panel) The **L-**STRF is estimated from **L** responses. It displays the average reference spectrotemporal responsiveness measurable with the (lower-frequency biased) speech stimuli of these experiments; this average speech spectrum is depicted by the side plot next to the rightmost panel. (Middle-panel) The **M**-STRF captures response selectivity during miming. Highlighted by the dashed circle is an apparent early wave of excitatory influences that precede the responses. (Right-panel) **S**-STRF exhibits strong suppressive influences (highlighted by the dashed circle) that are potentially responsible for the decrease in auditory responses during speaking.

Figure 3 illustrates the method and the findings from all 4 subjects. Further details of the procedures and data analyses are available in Methods. The first step is to “train” the inverse mapping function G_**M**_ between the **M** responses from all auditory electrodes to the spectrograms of corresponding stimuli. This G_**M**_ is then used to reconstruct the same stimuli from all other unseen **L**, **S**, and **N** responses. If any of these responses share similar spectrotemporal modulations with **M**, then the reconstructed spectrograms should reflect this similarity. The same rationale has been successfully applied in other cortical recordings, such as in vision (Haynes and Rees 2005; Reddy et al. 2010; Horikawa et al. 2013) and speech and music (Martin et al. 2014, 2018). Figure 3*A* explains the procedure and illustrates an example of a speech sentence and its corresponding reconstructions from **M** (using the trained filter G_**M**_), as well as **L**, **S**, and **N**. As expected, the reconstructions from the **M** training data are the most correlated with the stimuli. However, the same G_**M**_ is also able to reconstruct spectrograms from the unseen **L** and **S** responses, albeit less accurately as the correlation measures indicate. The reconstructions from **N**, by comparison, are worse as these responses have no stimulus-induced activity. A summary of comparisons from all responses in the 4 subjects are shown in Figure 3*B* (as well as for each subject separately in Supplementary Figures 3.1**–****3.9**). Across all stimuli and all subjects, the reconstructions from **L** and **S** responses correlated more strongly with matched **M** reconstructions than the reconstructions from N responses did (*P* < 0.001, 2-sample *t*-test). This suggests that the mapping function, trained solely on **M** conditions (G_**M**_), captured both the spectral and temporal features shared with the **L** and **S** conditions. For the temporal features, these results are consistent with the findings in Figure 2 which already confirmed the significant “temporal” correlations between the response waveforms. For the spectral features, their fidelity is confirmed by noting that if we randomize the spectral channels of the stimulus spectrogram or its reconstructions, then all the correlation distributions in Figure 3*B* collapse to around zero (i.e., completely overlapping the **N** distributions). This indicates that the temporal correlations are only significant between the corresponding spectral channels. Therefore, we conclude that the reconstructions, and hence the original **M, L,** and **S** responses, preserve the spectrotemporal features of the stimuli.

To assess further the within-stimulus fidelity/reliability of the reconstructions across each condition, we used the ranking method described earlier in Figure 2*B* in which the correlation between each reconstructed spectrogram and its corresponding original stimulus was ranked relative to the correlations with all other 60 stimuli (normalized between 0 and 1). The average ranks from all such matches are indicated by the different color arrows in Figure 3*C*, relative to the distribution of random rankings that result from shuffling all stimulus labels. When trained on the **M** responses, the reconstructions of **L** and **S** (but not **N**) had higher average ranks than would result from random assignments. This further supports the idea that the reconstructed spectrograms from **L** and **S** (using the G_**M**_ mapping) meaningfully reproduced the original spectrograms. Curiously, as was the case in other comparisons thus far, **S** responses appear to share less with **M** than those during listening **L**, perhaps because of ongoing interactions between auditory and motor influences during speaking (as we discuss later).

In another way to characterize the reliability of the spectrotemporal character of the responses, we performed **K**-fold cross-validation of reconstruction filters on segments of the responses within each condition. This method does not compare responses from different conditions against each other but rather assesses the reliability and predictability of each condition individually. For example, **M** responses were arbitrarily divided into **K** segments; reconstruction filters G_**M**_ were then trained on a subset of the data (**K-1** segments) and used to predict the spectrogram of the remaining **K**th segment, which subsequently was correlated with the corresponding **K**th segment of the stimulus spectrogram. The **K**-fold cross-validation procedure was repeated for each response condition, resulting in a distribution of correlation-coefficients for **L,S,** and **M** conditions. These distributions were each plotted against the distribution generated from **N** responses, as noise condition responses are presumed not to have a predictive structure. Figure 3*D* illustrates the results obtained from the average of all subjects (individual subject results are available in Supplementary Figures 3.1**–****3.9**). In all cases, we found a difference between the distributions of **L**, **S**, and **M** versus **N** (***P*** < 0.001, 2-sample *t*-test), with the largest difference in the **L** responses, reflecting their larger and deeper modulated structure. The correlations were smallest for the **M** condition but still significantly different from those of the random **N** responses (***P*** < 0.01**,** 2-sample *t*-test).

Finally, to gain further insights into the dynamics and spectral character of the **M**, **L**, and **S** responses in relation to the speech stimuli, we computed the spectrotemporal receptive fields (**STRF**s) for each condition (Klein et al. 2000), averaged from all the subjects and electrodes, as shown in Figure 3*E*. One rationale for this global measure is that in each of these experimental **M**, **L**, and **S** conditions, the stimulus drove the auditory electrodes through different routes and engaged diverse local processes, and hence the transformations from the spectrogram to electrode responses can be efficiently approximated and interpreted through their STRFs. The STRFs were computed for each electrode by estimating the response-prediction filters (using the cross-validation method described above; see Methods). Each STRF was then weighted according to its prediction reliability (or the correlation coefficient between predicted and actual responses), and all resulting (weighted) STRFs were then averaged over electrodes and subjects. Finally, to confirm the reliability of the STRFs, we verified that they remained unchanged if they were computed using a smaller portion of each stimulus and response (e.g., by removing 100 ms at the onset of each stimulus and corresponding response). However, the STRFs became randomly shaped and insignificant as expected when computed after scrambling the order of the stimuli relative to their responses.

It is well-known that cortical STRFs measured during listening **L** to speech stimuli vary considerably in the details of their tuning, polarities, latencies, and locations (Elhilali et al. 2007; Mesgarani et al. 2014; King et al. 2018), and hence the average **L-**STRF (left panel; Fig. 3*E*) indicates that, while responses were evoked at all frequencies (dashed circle; Fig. 3*E*), they were strongest at low frequencies (~200 Hz), a preference that is also seen in the other STRFs, likely reflecting the frequency-bias of the speech stimuli themselves (Fig. 3*A*).

Important other details, however, are revealed when considering the **M**- and **S**-STRFs. For example, the **M**-STRF (middle panel Fig. 3*E*), measured during the silent motion of the vocal tract, indicated that electrodes were activated 50–100 ms prior to the onset of the responses. As we shall discuss later, this may reflect pre-motor activity inducing “predictive” responses in the auditory-electrodes as postulated by the forward pathway (Fig. 1*A*). In contrast, the **S-**STRFs which broadly resembled the tuning and shape of the **L**-STRF displayed a large early wave of inhibition, which may explain the suppressed responses often measured during speaking or vocalization (Houde et al. 2002; Eliades and Wang 2003).

In summary, the results from the various analyses in Figures 2 and 3 lead to the same conclusion—that the **M** responses induced in auditory electrodes during silent miming exhibit spectrotemporal details comparable to those evoked during listening (**L**) and speaking (**S**). Responses during **M** are therefore not a broad “static” influence (possibly suppressive) on the speech responses during listening and speaking, but rather are rapid and spectrotemporally similar to, although significantly smaller than, the speech responses during listening and speaking.

### Spectrotemporal Specificity of Motor-Electrode Responses Induced by Sound

So far, we have focused on the motor influences through their presumed forward projections into the auditory responsive regions. Equally important are the inverse projections from the auditory cortex to the motor areas, as postulated in the structure of the MirrorNet (Fig. 1*A*). In the vocal-tract context, these inverse projections are known to induce motor neural responses during listening to speech (Wilson et al. 2004), but it is unclear if they are spatiotemporally detailed, or if they are similar to what would have been produced during utterance of the speech, a hypothesis reminiscent of the “Motor Theory of Speech” (Liberman et al. 1967). A key prediction of this hypothesis is that the motor activity should be temporally agile and commensurate with what is needed to move the articulators to produce speech. To test this idea, we compared the temporal structure of the motor-electrode responses evoked during speaking (**S**) and miming (**M**), with that measured during passive listening (**L**) to the same speech. The goal was to determine if the motor responses measured during listening were distinctive enough to reflect accurately the corresponding sentences that evoked them.

### Correlating Motor-Electrode Responses across Different Conditions

As described in Figure 1*B*, motor electrodes were defined as those that responded strongly during miming **M**. None of these electrodes responded appreciably during listening, and certainly none sufficiently enough to exceed the auditory-electrode selection criteria applied (Fig. 1*B*). Consequently, auditory and motor electrodes were mutually exclusive sets of electrodes in all subjects (anatomical positions are discussed in detail later). Motor electrodes were most active during silent miming (**M**), becoming slightly suppressed to 71% of **M** response (*r.m.s.*) power during speaking **S**, an analogous pattern to that seen on the auditory electrodes where auditory responses were also suppressed during speaking compared with listening. On the motor electrodes, **L** responses were weak (54% of **M)** but were still significantly larger than **N** (31%)**.**

**Figure 4:**
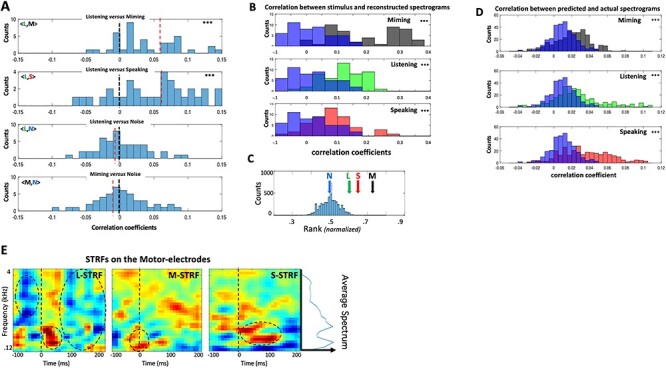
Analyses of responses on motor electrodes. (*A*) (Top panels) Distribution of correlation coefficients between **L** versus **M** and **S** responses, aggregated from all electrodes and subjects. Both are significantly positively shifted indicating shared response temporal structure among these conditions. (Bottom panels) Distributions of correlation coefficients between **N** versus **L** and **M** responses. **N** is not expected to share any structure with responses from the other conditions, and hence they are scattered around zero. The top two distributions are significantly shifted upwards relative to the bottom two (*P* < 0.001; 2-sample *t*-test). (*B*) Distribution of correlation coefficients between stimulus spectrograms and their reconstructions using G_**M**_, applied to **M**, **L**, and **S** responses. They are all significantly positively shifted relative to those reconstructed from **N** (in blue). (*C*) Average rank-ordering of correlations between each reconstructed spectrogram and its corresponding stimulus is significantly better than chance for **M**, **L**, and **S** conditions (5.7**σ**, 3.4**σ**, and 2.12**σ**, respectively) compared with chance for **N**. (*D*) Distribution of prediction correlations for **L**, **M**, and **S** are significantly shifted relative to those derived from **N** responses. (*E*) Average STRFs in three response conditions measured on all motor electrodes in three subjects. The dashed circles highlight excitatory and inhibitory features that are discussed in the text. The average speech spectrum is depicted by the side plot next to the rightmost panel.

Figure 4 illustrates a series of analyses and results that are analogous to those discussed earlier for the **M** responses on the auditory-electrodes in Figures 2 and 3. In Figure 4*A*, the detailed temporal structure of the **L** responses was compared directly across conditions through pairwise-correlations on each motor-electrode, pooled from subjects 1, 2, and 3. The top two panels demonstrate a significant positive bias in the correlations between **L** versus **M** and **S** indicating that, despite the absence of any articulatory motion during listening, there was neural activity on the electrodes that shared a similar temporally modulated structure with the responses during articulation in **M** and **S**. No systematically significant correlations were found between any of the responses versus the **N** condition.

### Reconstructing Spectrograms from Motor-Electrode Responses

The results above suggest that the inverse pathway induces responses on the motor electrodes during listening (**L**) that are phase-locked and somewhat similar to those evoked during articulating speech (Fig. 4*A*). However, it is unclear what exactly these responses represent. Thus, despite the known representational complexity of speech in the auditory cortex (Chi et al. 2005; Elhilali et al. 2007; King et al. 2018), it was nevertheless possible to reconstruct the spectrograms of the stimuli from the responses in order to interpret global features of the responses (Fig. 3*A*). Responses on the motor electrodes are likely related to vocal-tract articulatory parameters reflecting muscular motion, and hence they are at best a very indirect correlate of the stimulus spectrograms. However, given the unknown nature of these parameters, and the enormous complexity of their representation (Bouchard et al. 2013; Chartier et al. 2018), we have chosen to bypass these details and instead reconstruct the spectrograms with the hope that they may still preserve global characteristics and relationships among the motor responses and the corresponding stimuli.

Reconstruction filters (e.g., G_**L**_ or G_**M**_) were trained on the **L** or **M** responses, and then applied to reconstruct the spectrograms from the responses in the other conditions, as detailed earlier (Fig. 3*A*). If all these responses shared a common structure, then the reconstructions should be better matched to the original spectrograms compared with the reconstructions from noise **N**. Figure 4*B* confirms this conjecture showing that the distribution of correlations between reconstructed and original spectrograms are significantly better during **M**, **L**, and **S** compared with **N**. Furthermore, the reconstructed spectrograms were sufficiently detailed to be better associated with their corresponding stimulus sentences (among ~60 samples), as demonstrated by the average ranking for responses in all conditions in Figure 4*C*.

The reliability of the motor-electrode responses was next assessed by training predictive filters on a subset of the responses within each condition, and then cross-validating them on an unseen portion of the same responses (as in Fig. 3*D*). The accumulated results from subjects 1, 2, and 3 are shown in the three panels of Figure 4*D* for the **M**, **S**, and **L** conditions versus **N**. Predictions were significantly better correlated with the stimulus for **M**, **S**, and **L** conditions than for the noise **N.**

Finally, Figure 4*E* depicts the averaged STRFs measured on all motor electrodes from the **M**, **L**, and **S** responses, exactly the same way as for the auditory electrodes (Fig. 3*E*). During listening and speaking, the motor electrodes **L**-STRF (left panel) and **S**-STRF (right panel) resemble each other except for a striking strong wave of inhibition in the **L**-STRF (top) that surrounds the onsets. Since the **L**-STRF was measured on the motor-electrodes in the absence of any articulatory (motor) activity, it is thus analogous to the auditory **M**-STRF (middle-panel in Fig. 3*E*).

Conceptually, the most important conclusion of the above analyses is that listening induces on the motor-electrodes a meaningful systematic response to speech (**L**-STRF), which shares a resemblance to the temporally modulated structure of the responses evoked during speaking (**S**-STRF) and miming. In the context of the Mirror Network schematic of Figure 1*A*, this result is consistent with the existence of an inverse (Encoder) pathway projecting from the auditory to motor responsive regions, analogous to the forward (Decoder) projection from the motor to auditory responsive regions.

### Electrode Receptive Fields and Locations

Response measures in the analyses above were based on combining information from all auditory or motor electrodes in order to generate a global estimate of the interactions and the encoding of auditory–motor information. Individual electrodes naturally do not contribute equally or in the same way to the overall measures. For instance, it is evident in the histograms of Figures 2*B* and 4*A* that there was a sizable spread in how different electrodes contribute to the overall correlations between **L** and **M** responses (<**L**,**M**>).

Auditory-electrodes exhibit diverse cortical STRFs when measured with speech stimuli (David and Shamma 2013), but these also depend on the behavioral conditions (Fritz et al. 2003; Mesgarani et al. 2009), on the stimuli (Valentine and Eggermont 2004; Gourévitch et al. 2009), and on the nature of responses used to measure them. For instance, auditory- and motor-electrode STRFs in Figures 3*E* and 4*E* changed during **L**, **M,** or **S** scenarios, likely reflecting the engagement of diverse interactions and processes when generating the responses in different conditions. It is therefore critical to ask where the sources and destinations of these influences are and how they are manifested on electrodes located in different cortical regions. We approached these questions by explicitly examining the patterns of reverse correlations among all electrodes under the different conditions.

In the first approach, we measured the reverse-correlation (“revcor”) patterns between auditory and motor-electrodes (Klein et al. 2000; Gourévitch et al. 2009). This measure “pretends” that responses on one set of electrodes act as stimulus (source or input) to responses on another set of electrodes. It thus provides a detailed and explicit estimate of the correlations between the two responses, which may well be (but clearly not necessarily) related to their interconnectivity.

**Figure 5:**
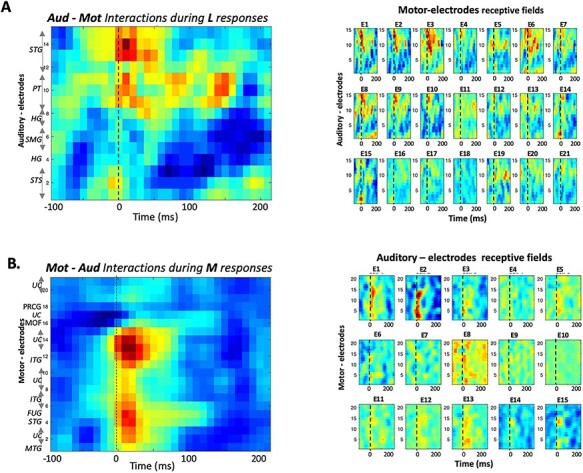
Interactions among auditory and motor-electrodes. (*A*) All data shown here are from subject 2. (Right panels) Reverse correlation of **M** responses on auditory and each of 21 motor electrodes, computes “auditory receptive-fields” that reveal the potential dependence of each motor-electrode responses on all the auditory electrodes. Each panel is weighted by its predictive ability to account for the responses on that electrode. (Left panel) The average of all motor-electrode panels reveals a selectivity to different auditory electrodes whose numbers and locations are identified on the *y*-axis. (*B*) (Right panels) Same as above except the reverse correlation is performed on the **L** responses on each auditory electrode relative to the activations from all motor electrodes. The resulting “motor receptive-fields” are weighted by their predictive ability. (Left panel) The average of all auditory-electrode panels reveals a potential selectivity to different motor electrodes whose locations are identified on the *y*-axis.

The small panels on the right in Figure 5*A* depict the revcor estimates from each of 21 motor electrodes in subject 2; these represent how motor responses during listening **L** are selectively and dynamically related to each of 15 simultaneously active auditory electrodes, whose indices and locations are indicated on the *y*-axis of the left-most panel. In effect, the panels display the “receptive field” of each motor electrode. All panels are shown on the same color scale, and each is weighted by its predictive reliability (see Methods for more details). The average of all the receptive fields is depicted by the large panel on the left in order to highlight the auditory electrodes most effectively correlated with the motor responsive regions of this subject. The strongest average correlations in Figure 5*A* emanate from auditory electrodes near **#9–#15**, which are all located in the secondary auditory fields of the PT and STG. The most reliably driven motor electrodes are **#1–3** and **#6–10**, which exhibit similar but gradually changing response dynamics and selectivity, and are located in or over the middle and inferior temporal gyri (Cheung et al. 2016).

Figure 5*B* provides an analogous, complementary view to the above interactions, instead treating the motor-responses during **M** conditions as “inputs” into each of the 15 auditory electrodes; the corresponding “receptive field” patterns are displayed in the 15 small panels. The most reliable auditory responses here appear on electrodes **#1–2** (STS) and #**14–15** (STG); these are most correlated with a cluster of motor electrodes near **#4** and **#14** located nearest to the STG and ITG. Finally, we note that in this subject, the HG electrodes did not apparently play a significant role in providing predictive or inverse responses between the auditory and motor responsive regions; and neither did the motor-electrodes located nearest to the primary motor areas in the PG (**#17–18**).

We have similarly analyzed data from two other subjects (Supplementary Figs 5.2  **and**  **5.3**). The broad outlines of the results are consistent with those already shown here. Specifically, the auditory electrodes from non-primary areas (STG, PT, INSULA, and STS) were the most related with the motor electrode responses during both **L** or **M** conditions, as is evident in the supplementary data of two more subjects 1 and 3. The one exception is the interactions depicted in the panel of Supplementary Figure 5.2 between an HG and motor electrodes during **L** responses. In motor electrodes, the interactions confirm the significant contribution of the MTG and STG (e.g., **#14** in subject 1 and **#13–16** in subject 3), as well as the absence of significant interactions from primary motor areas (e.g., postcentral gyrus **#12–13** in subject 1; and PG **#21** in subject 3).

Finally, our subject 2 was bilaterally implanted (Fig. 1*B*), and so we redid the analyses separately for the right and left hemisphere auditory and motor electrodes. We confirm here that both hemispheres reproduce the same findings reported earlier. This result is consistent with the findings reported by Cogan et al. (2014) on the bilateral nature of the sensorimotor responses in the cortex.

### Sensorimotor Interactions and Learning in the Mirror Network

Our findings thus far have addressed the first aim of this experimental study, characterizing the spectrotemporal specificity of the forward and inverse projections of the conceptual network presented in Figure 1*A*. We now address their functional significance, specifically in the context of speech production and perception, but more generally in enabling sensorimotor tasks. Developing and simulating a mathematical model of the Mirror Network highlights a potentially critical function of the forward projections, namely, to enable learning the inverse maps needed for control and performance of sensorimotor tasks.

**Figure 6:**
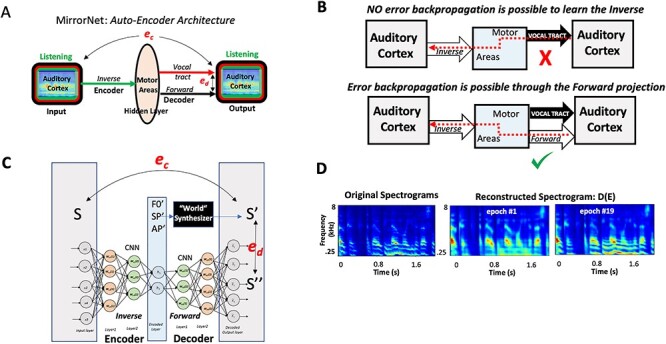
Simulating learning in the Mirror Network. (*A*). The overall layout of the sensorimotor interactions. It emphasizes the relative contributions of the inverse (Encoder) and forward (Decoder) projections between the auditory and motor areas. The overall network resembles a classic auto-encoder network that maps the auditory cortex activity onto itself through a hidden layer (motor regions), but with an additional non-neural motor-plant (vocal-tract) pathway that shares with the forward projection its motor input and auditory output. Two sources of error are available to train the neural pathways of the Encoder (**e**_**c**_) and Decoder (**e**_**d**_). (*B*) The critical role of the forward projection in providing a neural pathway for the (**e**_**c**_) error to backpropagate to the motor regions (hidden layers) so as to train the Encoder weights. (*C*) The MirrorNet implementation employs multiple layers of a convolutional neural network, and the “World” synthesizer as a simplified model of the vocal tract. (*D*) Training the MirrorNet results in progressive improvements in the reconstructed spectrograms projected through the sequence of Encoder–Decoder layers. The training is rather limited here involving only about 40 min of speech beyond the initialization with the random patterns.

We begin with a redrawing of the network of Figure 1*A*, by unfolding the inverse mapping from the forward as shown in Figure 6*A*, referred to henceforth as the *MirrorNet*. Here the auditory cortex is depicted twice, as an input and as an output. This organization of the system is well-known in the neural network literature as an **Auto-Encoder**, where the input (responses in the auditory cortex) is mapped onto itself at the output, through two transformations: an Encoder to a latent (hidden) representation (the motor responsive region here), and then through a Decoder back to the output (auditory cortex). Normally, such auto-encoder networks are simply trained by requiring that the Encoder and Decoder projections be able to reproduce the input with minimum error. In doing so, the auto-encoder finds a new, possibly more compressed and efficient but equivalent, representation of the auditory input as activations in the hidden (motor) region, which can still be mapped back to the auditory representations.

First, we consider learning the Decoder projections in the MirrorNet. In the sensorimotor literature, it has always been assumed that the forward predictive projection from the motor to sensory areas serves to monitor task performance, and to provide rapid feedback of errors to ensure accurate motor execution (Wolpert and Ghahramani 2000). This predictive role has also found extensive support theoretically and experimentally in the sensory perceptual domain (Keller and Mrsic-Flogel 2018). The formation of this projection in sensorimotor systems is conceptually straightforward in that it serves as a model of the motor plant and hence can be learned by minimizing the differences (***e***_***d***_) between the Decoder and vocal-tract outputs as illustrated in Figure 6*A*.

The inverse projection (or Encoder), on the other hand, serves to map sensory expectations and intentions into the motor commands necessary to reproduce them. However, learning a functioning inverse projection presents a significant challenge, for without a large set of predetermined exemplars (training data) to associate sensory signals to the correct neural motor commands, one has to resort to trial-and-error approaches. Classifiers and neural networks require large amounts of training data for accurate performance and generalization to unseen data, but it is often difficult to acquire such training material. For example, in the case of controlling the vocal tract, learning to pronounce words of a new language relies not on finding out what the motor commands ideally need to be (which is impossible!), but rather on listening to our pronunciation of the words and trying to map the perceived errors (***e***_***c***_) back to implicit corrections of the motor commands. As illustrated in the top panel of Figure 6*B*, this backward propagation of the error to the motor areas conceptually requires the inverse of the vocal tract to be computed so as to translate the sensory errors into motor-command adjustments that subsequently can be optimized by adjusting the inverse mapping. In general, computing the vocal-tract inverse is difficult if not impossible because of its complexity, nonlinearity, and our incomplete knowledge of its workings.

The MirrorNet in Figure 6*B* (bottom panel) solves this problem by adding a forward projection that serves as a parallel, “neural” model of the vocal tract**.** The critical value of this “neural” projection is that it readily provides a route for the ***e***_***c***_ errors to backpropagate to the motor areas, enabling training of the inverse mapping. Figure 6*C* illustrates a schematic of the resulting auto-encoder network, which learns its connectivity by backpropagating the error (e.g., ***e***_***c***_) through its “neural” pathways from stage to stage, adjusting the weights as the error proceeds backwards. The MirrorNet learns its Decoder weights by minimizing ***e***_***d***_ as discussed earlier; notably, the *Encoder* is also learned by backpropagating to minimize the error (in this case, ***e***_***c***_) through the *Decoder* neural pathway. Without the Decoder forward projection, the Encoder *inverse* mapping cannot be readily learned in this way since the error ***e***_***c***_ has no route to propagate backwards through the motor plant.

We thus conclude that a crucial role played by the forward projection is to provide a pathway to learn the inverse mapping in an unsupervised way, and without any need for explicit motor training data. That is, by simply listening and uttering the words, the errors are automatically used to guide the vocal tract to reach its sensory target.

### Simulating Learning in the MirrorNet

A brief demonstration of “unsupervised” learning in the MirrorNet is provided here to illustrate the critical role of the forward projection in facilitating the learning of the *inverse* mapping. The MirrorNet shown in Figure 6*C* is implemented in *PyTorch* with convolutional layers modeling the Encoder and Decoder pathways (see Methods for details). For the (input and output) auditory representations, we computed the auditory spectrogram, a representation mimicking the cochlear outputs (Chi et al. 2005; Mesgarani et al. 2006). The vocal-tract model was simulated by the “World” synthesizer (Morise et al. 2016), a widely used tractable vocoder model that takes three sets of input parameters as a function of time to synthesize a speech waveform: a spectral envelope function (SP), a pitch track (F0), and voicing/non-voicing indicator signals (AP). The goal of the MirrorNet here was to iteratively learn the Encoder weights (starting from random initial values) that map any (input) auditory spectrogram to the “motor” parameters that would both 1) reproduce the same spectrogram through the “World” synthesizer and also 2) simultaneously regenerate it at the output of the Decoder projection, in which case both errors ***e***_***d***_ and ***e***_***c***_ are minimized.

The network was initialized with random *Encoder* and *Decoder* weights that were fully trained using <60 min of speech. Two important procedures speeded up and guided the learning of the correct mappings: 1) an initialization training epoch in which the network was briefly trained to minimize only ***e***_***d***_ using random synthesizer-like parameters SP, AP, and F0 and 2) training the Encoder and Decoder alternately. The initialization epoch guided the Decoder toward reproducing the same type of output spectrograms as the synthesizer does, even if the input activations (in the hidden layer) were random. The alternating training procedure for network weights was as follows: the Decoder (resp., Encoder) weights were trained with epochs in which only ***e***_***d***_ (resp., ***e***_***c***_) is minimized via error backpropagation while the Encoder (resp. Decoder) weights remained fixed. These procedures succeeded in training the MirrorNet in an unsupervised manner with normal speech material, thus demonstrating the utility of the forward pathway in learning the task of driving the synthesizer. Figure 6*D* illustrates how reconstruction errors decreased over training epochs, evident in the improvement in quality of the reconstructed speech spectrograms of an unseen sample sentence over epochs. Further technical details of constructing and training this neural network are given in Methods.

Once the network was trained, it could readily inverse-map its sensory inputs (speech in this case) to the necessary parameters that drive the associated motor plant (vocal tract). Furthermore, the forward projection could still participate in its other commonly proposed predictive and control roles as a model of the motor-plant. The MirrorNet structure therefore is sufficiently general to serve as a model for analogous sensorimotor tasks requiring learning of a skilled performance, like playing a musical instrument, reading and writing, or training an autonomous vehicle to navigate traffic.

## Discussion

We begin by summarizing the findings and conclusions of our experiments and computational simulations, and then describe their implications for our understanding of sensorimotor interactions, especially for learning to control sensorimotor tasks.

First, we confirmed the projections postulated to exist in the network of sensorimotor interactions as in Figure 1*A*. Recordings during silent miming (**M**) revealed measurable responses in auditory responsive regions, confirming the influence of presumed forward projections from the motor areas to the auditory-responsive cortex. During listening (**L**) without any motor actions, significant responses were also measured in the motor areas confirming the existence of an inverse projection. Finally, responses during speaking (**S**) were found to be, as previously reported, suppressed relative to the **M** and **L** responses in motor and auditory responsive regions, respectively.

Second, detailed analyses of signals carried by the forward and inverse projections revealed remarkable spectrotemporal specificity, sufficiently adequate to encode individual sentences. Thus, during a skilled task like speech production, we conjecture that these auditory–motor interactions modulate and control auditory and motor responses in detailed and meaningful ways so as to play a role in learning and performance of the auditory–motor tasks.

In the experiments and analyses reported here, the forward and inverse activations (**M** in auditory and **L** in motor electrodes) were small because they were measured in the absence of other background responses due to acoustic or motor stimuli. Consequently, to demonstrate the meaningful interpretation of these responses, we had to apply diverse methods, for example, spectrogram reconstructions, STRF predictions, and correlation rankings, all with varying degrees of confidence. However, in the case of speaking, **S** responses in both auditory and motor electrodes are substantial, and they are strongly modulated by inputs projected from the counter regions. This was best demonstrated by the large changes between the various average STRFs in Figures 3 and 4, for example, the changes from **L**-STRF to **S**-STRF to **M**-STRF in Figure 3*E*.

Specifically, STRF changes revealed remarkably different dynamics and patterns of interactions depending on the task that complement the interpretations gained from the direct response measurements. For instance, when speaking (**S**), relatively strong inhibitory influences are seen in the **S**-STRFs preceding the onset of the responses. This timing seems to coincide with a preceding wave of responses on the **M**-STRFs. One possible interpretation of these patterns is that the early **M** activation reflects responses of local recipient inhibitory interneurons and that these in turn exert their inhibitory influences during speaking when the evoked auditory responses are sizable. This interpretation is also consistent with the fact that auditory **L** responses (which presumably supply no motor inputs) do not exhibit either of the preceding waves of activation in the **L**-STRFs. Motor electrodes, on the other hand, receive an inhibitory wave preceding the **L** responses (**L**-STRFs) that roughly coincides with an early activation of the **M** responses (**M**-STRF). The **S** responses which combine motor and auditory interactions are complex and less punctate, perhaps reflecting the local interactions between the **M** and **L** sources. All these details remain to be addressed in future analyses that would consider the timing of the interactions (e.g., Cogan et al. 2014; Liebenthal and Möttönen 2018), especially on individual localizable electrodes.

Third, the high spatiotemporal resolution of the ECoG allowed us to localize sources and destinations for the auditory–motor interactions and to reveal their relative timings. The results on the whole are consistent with findings from global imaging data with fMRI, EEG, and MEG. For instance, we found that the forward and inverse projections are largely between non-primary auditory responsive regions such as the STG, PT, versus MTG, ITG on the motor side. Non-primary regions are known to be far more plastic and hence susceptible to the effects of behavioral engagement and learning from experience.

Finally, to demonstrate the functional significance of the forward projections in the context of learning of skilled auditory–motor tasks like speaking and musical playing, we simulated the structure of the MirrorNet and showed how it can acquire the skill needed to control a motor-plant like the vocal tract. The key insight is that the forward path, long postulated to be the route of predictive responses needed for vocal control and perception, can instead play a different role, that of a neural conduit to backpropagate errors between the produced and received speech, which are necessary to learn the *inverse* mapping from the auditory to the motor responsive regions. While hugely simplified, this computational model still plainly demonstrated the principle that without the forward neural pathways, learning of a skilled motor task like speaking becomes an unwieldy trial-and-error procedure.

We next discuss the implications of these findings for the theories of sensorimotor interactions in the particular context of speech production and comprehension, and more broadly with respect to sensory prediction and the hypothesized function and significance of the mirror neurons. We end with a brief recount of the functional significance of forward projection in learning the inverse mapping, and how this idea provides a general solution to the more general problem of learning how to control and monitor performance of complex motor-plants.

### Sensorimotor Interactions in Nonhuman Animals

The experimental findings that justified the functional role of direct interactions between sensory percepts and motor acts are extremely diverse, beginning with the notion that a corollary discharge can function as a filter that suppresses self-generated sensory input allowing the animal to remain sensitive to external stimulation (Poulet and Hedwig 2006), to stabilize visual receptive fields by predicting saccade targets (Sommer and Wurtz 2002), to suppress auditory cortical activity during locomotion (Nelson et al. 2013; Schneider et al. 2014), or to facilitate vocal learning in birds (Prather et al. 2008; Keller and Hahnloser 2009). Aside from the corollary discharge, or the forward projection common to all these examples, there are fundamental differences among them. For instance, all except for the last example are due to instinctive processes that are not learned the way it is with the projections in birds learning a vocal repertoire. Therefore, we shall distinguish and refer in our commentary here only to skillful continuous sensorimotor actions requiring extensive practice such as the control of the vocal tract in speech production or of the hand and fingers in musical playing. Hence, neither of these sensorimotor interactions is expected to exist with untrained motion or inappropriate sounds, as was demonstrated for speech and vocal tract production in Cogan et al. (2014).

At the phenomenological level that we adopt in this study, vocal learning in birds bears a close resemblance to the basic structure of human vocal-tract control and learning (Fig. 1*A*). I physiological single-unit recordings in birds have unambiguously established the analog of the forward pathway, that it likely generates a detailed spectrotemporal representation of the stimulus which mimics that received from the ear during vocalizations (Prather et al. 2008), and that this in turn would allow the bird to compare them and minimize the difference, and hence learn how to control its vocal source (Keller and Hahnloser 2009). Even the hypothesized induction of auditory responses with silent “chirping” seems to have been mentioned in passing many decades ago (Williams and Nottebohm 1985)! All these details are reminiscent of the two directional projections and minimization of errors *e*_***d***_ and *e*_***c***_ depicted in Figure 6.

### Relation of the MirrorNet to Theories of Speech Perception and Production

Sensorimotor interactions have long been known to play a key role in promoting skilled task performance, and there is especially a substantial body of experimental studies and theoretical models of how the sensory and motor domains are linked during speech perception and production. These models vary considerably in their levels of description and details. Some have focused on analytical formulations of the processes needed to control vocal-tract dynamics in speech production (Tourville et al. 2008; Houde and Chang 2015; Parrell et al. 2019). Others provided descriptions that encompass large regions of the brain combining both speech production and comprehension, and postulating specific bilateral neural substrates and connectivity patterns among them (Hickok and Poeppel 2007; Poeppel et al. 2012; Cogan et al. 2014; Poeppel 2014). Anatomically grounded accounts have also emerged from imaging experiments with fMRI and EEG that have emphasized the overall bidirectional flow of information across motor and sensory regions and that have attempted to situate these processes within the overall flow of information from the auditory to the prefrontal cortex (Rauschecker and Scott 2009; Lima et al. 2016). The study by Cogan et al. (2014) comes closest to our experimental methodology in its recordings of responses in the **M**, **L**, and **S** conditions in similarly defined auditory and motor electrodes. However, all their analyses had concentrated on the strong overt auditory and motor responses and the **S**-responses, and not as we do, on the covert activations due to the forward and inverse projections that are also evident in their data (e.g., their Fig. 2*D* displays weak AUD (green) and PROD (blue) responses during opposite conditions).

In contrast to previous accounts of sensorimotor interactions, the MirrorNet schematic that frames our experiments and motivates the data analyses is strictly phenomenological in flavor. Thus, while the postulated processes and interactions are biologically plausible and supported by experimental evidence, the network model is largely agnostic with respect to the specific anatomical regions that source or receive the forward and inverse projections; the biological implementations of the error signals; or how they might be backpropagated to adjust the weights and learn the projections. The network, however, makes specific predictions that intersect and potentially impact other proposed formulations. For instance, the sensorimotor inputs into the auditory and motor cortical regions are evidently rapid, with dynamics that are commensurate with those of speech and the movements of the vocal tract. Furthermore, they are encoded in a manner consistent with the representational domain of the recipient region, that is, the forward projections are auditory, and the inverse projections are motor (Fig. 6*A*). The projections are also likely to be quite adaptive so as to learn (forward) and control (inverse) the specific structure of a person’s vocal tract (Houde and Jordan 2002). Hence, these properties are consistent the finding that the most auditory and motor electrodes implicated in the sensorimotor projections were localized in secondary (auditory) areas like the STG and PT (Fig. 5), and non-primary motor areas. These auditory responsive regions are highly adaptive, task-dependent, but also spectrotemporally rich and agile to allow for reliable speech representation (Mesgarani et al. 2014), properties that are consistent with the MirrorNet requirements.

In an extensive excellent review of speech perception and production theories, Skipper et al. (2017) distilled and contrasted a few of the most salient of these ideas. To summarize, at one extreme, the “Motor Theory of Speech” argues that speech perception is firmly anchored in a motor (articulatory) representation of the signals (Liberman and Mattingly 1985). On the other extreme, the neurobiologically based “dual stream” model dissociates the two domains into two streams, with one (ventral) postulated to serve speech perception and recognition, while the other (dorsal) controls speech production (Hickok and Poeppel 2007). The Analysis-by-Synthesis model is intermediate between the above two theories, advocating a more nuanced “constraint” on speech perception by the motor commands of the vocal tract that produce it (Poeppel and Monahan 2011; Stevens and Halle 1967).

At first glance, the MirrorNet structure (Fig. 6*A*) seems to be consistent with all these theories. Thus, to begin with, the Encoder branch maps auditory responses to the motor (vocal-tract) domain, analogous to the Motor Theory of Speech, while the Decoder projection transforms motor commands to auditory representations implementing the speech production implied by the dorsal stream of the dual stream hypothesis. Furthermore, mapping signals in the MirrorNet to and from the auditory and motor regions implies that the resulting representations (be it articulatory commands or auditory responses) must be highly constrained so as to be consistent across them, much as postulated by the Analysis-by-Synthesis model.

However, these correspondences become more interesting and intricate if speech production is considered to be more than simply executing articulatory commands issued in “motor areas” to produce auditory responses. Rather, for speech production, these motor areas must be intimately linked to brain regions where abstract concepts are first transformed into linguistic forms through access to the lexical and phonetic stores, before being converted to the appropriate corresponding articulatory commands. Therefore, one has to conclude that the “motor areas” of Figure 6*A* are in fact part of an extended distributed network of regions across large parts of the brain. And consequently, for these “motor-linguistic” regions to be at the terminus of the Encoder projection of the MirrorNet (Fig. 6*A*) allows this pathway to serve speech comprehension exactly as postulated by the ventral-stream hypothesis.

In summary, it is evident that if the “motor-areas” in Figure 6*A* are viewed as part of a distributed set of sensorimotor brain regions that participate in the many processes involved in speech comprehension and production, then the overall structure of the sensorimotor MirrorNet and its plasticity during learning strongly supports a seamless link between speech perception and production, albeit with substantial transformations from the sensory to the motor modalities that are both learned and constrained by experience.

### Beyond Speech Perception and Production

The framework of the MirrorNet is quite general and can serve many contexts outside of speech production and the vocal tract. Any highly practiced actions associated with the reception or production of sensory signals would be served well by such a network as a means for controlling the motor-plant and learning its commands. For instance, sign language and lip reading are identical to speech production and perception in the context of the MirrorNet, but with visual and proprioceptive signals replacing the auditory, and hands, arms, or lips replacing the vocal tract. Another example is playing the violin, which involves extensive training of the fingers, arms, and postural musculature—the motor-plant—to produce the music. Forward projections must learn gradually with practice to model this motor plant. Simultaneously, the *inverse* projection adapts to map the desired music into motor commands, and the learning thus proceeds by minimizing the two errors (Fig. 6*A*). Therefore, the MirrorNet structure predicts that these projections are highly specific to the skilled task that trained them, and hence their activations would not be recruited by inappropriate actions and irrelevant sensory signals, as was demonstrated by the speech selectivity reported for vocal-tract activations (Cogan et al. 2014).

In fact, MirrorNet interactions need not involve a motor task or motor-plant at all, but rather any constrained transformation that is not significantly amenable to adaptation. For instance, reading or sounding out a text is a transformation of a visual image (text) into corresponding sounds, often with complex rules of phonation (analogous to the complex rules of moving the vocal tract) (Slowiaczek and Clifton 1980). The forward projection would gradually learn the rules for mapping text to sounds, and in time, sound becomes an “imagined” output or the meaning of the text. The inverse mapping from the sound provides the image of the “expected” text—an imaginary writing task. These designations of course can be altered to describe learning to write or draw from a visual or an auditory image.

Therefore, the key idea common to all the above scenarios is an auto-encoder network with forward and inverse mappings (Fig. 6*A*), which is the essence of the idea of the “mirror neurons.” However, many extraneous issues have been appended to this network that are not an essential part of its function and that have led to numerous criticisms (Lotto et al. 2009; Hickok 2014). For instance, consider the role of the forward projection, which has been widely assumed to provide a predictive signal (the “efference copy”), to facilitate control of motor performance (Wolpert et al. 1995), or to provide a sensory goal rather than a precise prediction (Caroline et al. 2013). However, it is also possible to argue that this projection serves primarily as a route for the backpropagation of the error needed to learn the *inverse* mapping, without which it is difficult to control the vocal tract. Therefore, the mirror neurons can serve an important function, but that does not need to include the “higher-level” cognitive tasks ascribed to them, from speech comprehension to empathy.

Finally, the architecture of the MirrorNet has been invoked in many perceptual contexts since it lends itself to many interpretations. One common case in point is as a substrate for imagination, that is, sensory percepts devoid of external stimuli or actions without actual movements (Tian et al. 2016). In the MirrorNet, the forward projection of a skilled pianist can recapitulate musical percepts by simply moving her fingers appropriately without actually producing a physical sound (Martin et al. 2018). In fact, as mentioned earlier, Martin’s study had already demonstrated that the “imagined” activity, which is experimentally similar to our **M** responses, exhibited detailed spectrotemporal structure much like the **L** responses. Similarly, the urge to dance or tap when listening to a beat or a melody can also be interpreted as commands injected from a trained inverse pathway into the appropriate motor areas. Such imagination can be recast as an expectation, anticipation, or prediction of sensory stimuli from a contextual memory or motor areas, and hence may serve a preparatory function (Persichetti et al. 2020). In fact, this view is consistent with Cogan et al.’s (2014) findings of sensorimotor transformations where auditory responses were shaped by *subsequent*, hence expected vocal-tract actions. The MirrorNet, therefore, can be seen as a unifying architecture that can harmoniously organize diverse perceptual processes and sensorimotor tasks.

## Notes

We acknowledge the valuable advice and suggestions throughout this project by Dr. Stephane Mallat, especially in regards to the MirrorNet section. *Conflict of interest*: None declared.

## Funding

National Science Foundation (#1764010 to S.S.); European Research Council (*Neume* to S.S.); National Institutes of Health (NIDCD, DC005779 to S.S., DC014279 to N.M.).

## Supplementary Material

Legends-for-Supp-Figs_tgaa091

Slide08_tgaa091

Slide09_tgaa091

Slide10_tgaa091

Slide11_tgaa091

Slide12_tgaa091

Slide13_tgaa091

Slide14_tgaa091

Slide15_tgaa091

Slide16_tgaa091

Slide17_tgaa091

Slide18_tgaa091

Slide19_tgaa091

Slide20_tgaa091

Slide21_tgaa091

Slide22_tgaa091

Slide23_tgaa091

Slide24_tgaa091
