# Supplementary material for: Learning Speech Production and Perception through Sensorimotor Interactions
Source: Cereb Cortex Commun. 2020 Nov 27;2(1):tgaa091. doi: 10.1093/texcom/tgaa091 (PMC7811190; doi:10.1093/texcom/tgaa091)
Supplement: Legends-for-Supp-Figs_tgaa091 [file legends-for-supp-figs_tgaa091.docx]

**Supp.Figure 1**

**(Left panel)** The correlation-coefficients between all pairs of auditory *and* motor-electrodes in Subject-2. All measurements involved the responses within each condition separately. On average, the correlations between the auditory and the motor sets of electrodes are quite weak compared to within each electrode set. This suggests that the responses across the two electrode regions are of a different nature, and hence are weakly correlated. See text for more details. **(Right panel)** Same as *left-panel* but for Subject-1.

**Supp.Figure 3.1**

Average correlation-coefficients between *original* and *reconstructed* spectrograms from responses in each of the **M**iming, **L**istening, and **S**peaking conditions, accumulated from all frequencies and electrodes for each of the 4 subjects. The correlation-coefficients for each subject are the mean of each condition (**M, L,** and **S**), and are to be compared to the **N** reconstructions. The **M**, **L**, and **S** mean correlation coefficients (leftmost 3 columns) are in often higher than those due to **N** responses (rightmost column), indicating better matches to the original spectrograms, and hence the presence of spectrotemporal structure related to the stimuli.

**Supp.Figure 3.2**

Subject-1 average rank order of the correlations between a sentence and its corresponding reconstruction, compared to all other sentence comparisons. In general, reconstructions from **M**, **L**, and **S** response conditions are usually sufficiently accurate to allow reasonable recognition of the corresponding stimuli with above chance accuracy, i.e., they are ranked higher than those due **N** responses. In this subject, the S responses were rather weak and similar to noise.

**Supp.Figure 3.3**

Subject-1 distribution of correlation-coefficients between original and reconstructed spectrograms based on training G**_M_**, G**_L_**, G**_S_**, G**_N_** filters on selected response segments, and cross-validated with predictions from unseen segments. **N** responses produce worse predictions than **M**, **L**, and **S** conditions.

**Supp.Figure 3.4**

Subject-2 average rank order of the correlations between a sentence and its corresponding reconstructions. Other details are as in **Supp.Figure 3.2**

**Supp.Figure 3.5**

Subject-2 distribution of correlation-coefficients between original and reconstructed spectrograms. Other details are as in **Supp.Figure 3.3**

**Supp.Figure 3.6**

Subject-3 average rank order of the correlations between a sentence and its corresponding reconstructions. Other details are as in **Supp.Figure 3.2**

**Supp.Figure 3.7**

Subject-3 distribution of correlation-coefficients between original and reconstructed spectrograms. Other details are as in **Supp.Figure 3.3**

**Supp.Figure 3.8**

Subject-4 average rank order of the correlations between a sentence and its corresponding reconstructions. Other details are as in **Supp.Figure 3.2**

**Supp.Figure 3.9**

Subject-4 distribution of correlation-coefficients between original and reconstructed spectrograms. Other details are as in **Supp.Figure 3.3**

**Supp.Figure 4.1**

(*Left-panel*) Average correlation-coefficients between *original* and *reconstructed* spectrograms from motor-electrode responses in each of the **M**iming, **L**istening, and **S**peaking conditions, accumulated from all frequencies, motor-electrodes and all 3 subjects. In each row, the correlation-coefficients are the mean of each condition (**M, L,** and **S**), and are to be compared to the **N** reconstructions. The reconstruction filters are different: G**_M_** in the first row, **G_L_** in the second row, and **G_S_** in the third row. In most cases, the reconstructed spectrograms are better matched to the original spectrograms (higher mean correlation coefficients) than those due to the noise N, indicating better matches to the original spectrograms, and hence the presence of temporal structure related to the stimuli.

(*Right-panel*) Same as *left-panel* except results are shown separately for the three subjects, all trained on the listening condition **G_L_**

**Supp.Figure 4.2**

Subject-1 distribution of correlation-coefficients between original and reconstructed spectrograms based on training G**_M_**, G**_L_**, G**_S_**, G**_N_** filters on selected response segments, and cross-validated with predictions from unseen segments. **N** responses produce worse predictions than **M**, **L**, and **S** conditions.

**Supp.Figure 4.3**

Subject-2 distribution of correlation-coefficients as in **Supp.Figure 4.2**

**Supp.Figure 4.4**

Subject-3 distribution of correlation-coefficients as in **Supp.Figure 4.2**

**Supp.Figure 5.1 & Supp.Figure 5.2**

Details are exactly as in Figure 5, except for subject-1 and subject-3.

**Supp.Figure 5.3**

Locations of most Auditory- and Motor- responsive electrodes in three subjects-1,2,3. The Auditory-electrodes are colored in yellow-brown; motor-electrodes are colored in light- deep blue. The numbers correspond to those used in **Figures 5** and also in **Supp.Figures 5.1& 5.2.**  The darker colors are those of electrodes with specific mention in the text and in reference to **Figure 5** and **Supp.Figures 5.1** & **5.2**.
