# Supplementary figures and images for: Learning Speech Production and Perception through Sensorimotor Interactions
Source: Cereb Cortex Commun. 2020 Nov 27;2(1):tgaa091. doi: 10.1093/texcom/tgaa091 (PMC7811190; doi:10.1093/texcom/tgaa091)

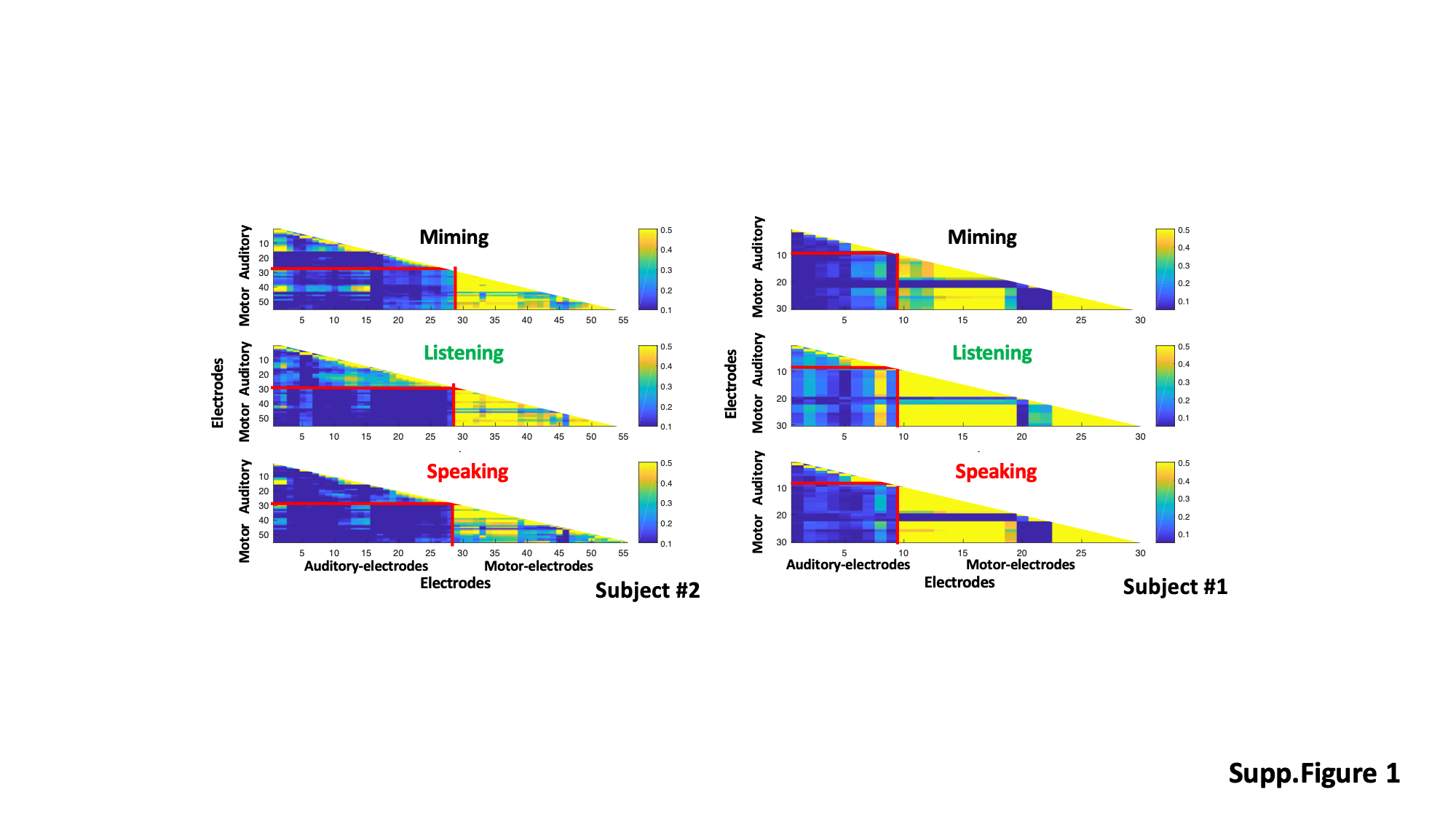

Supplement: Slide08_tgaa091 [file slide08_tgaa091.zip › Slide08_tgaa091.tiff]

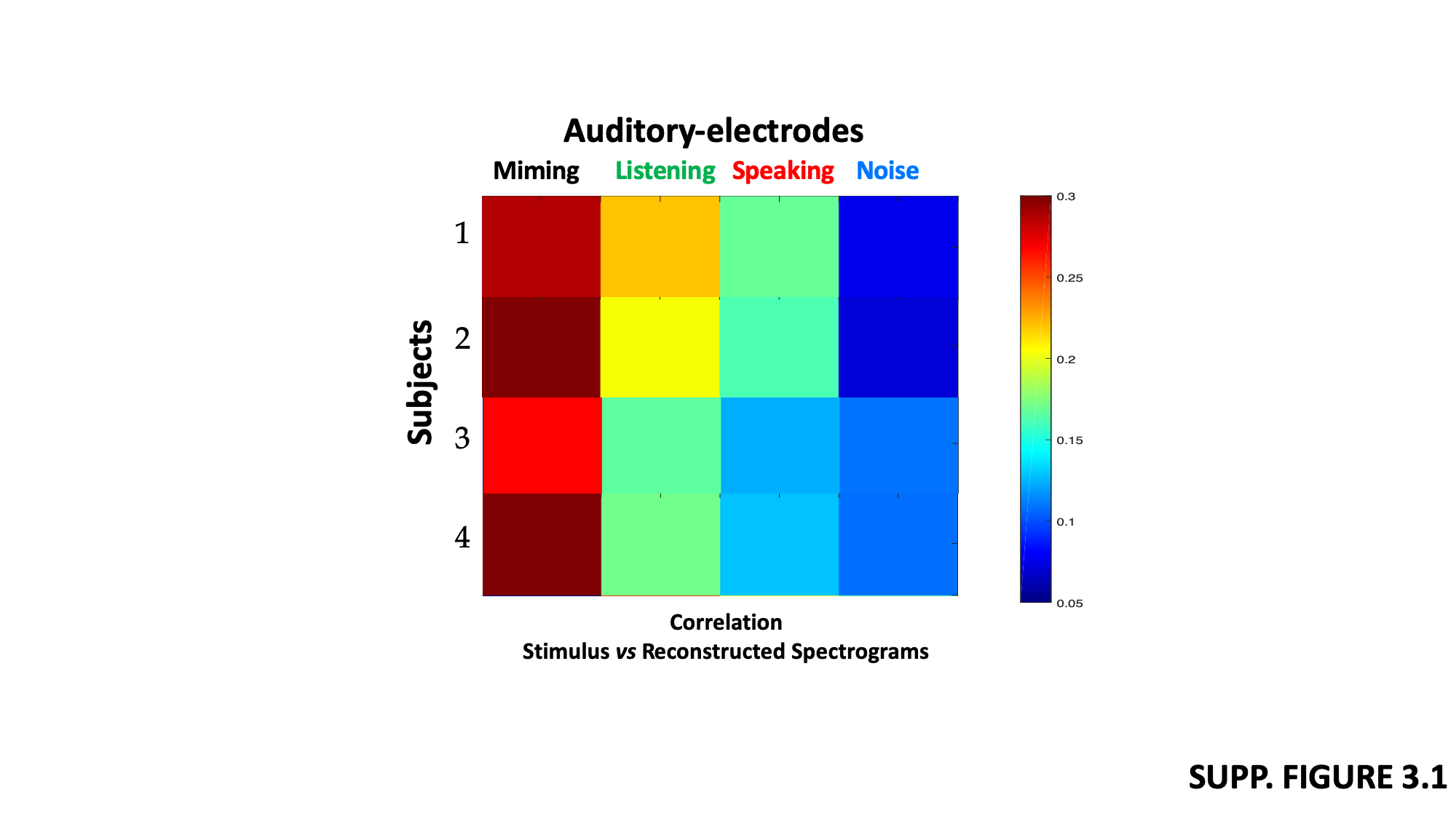

Supplement: Slide09_tgaa091 [file slide09_tgaa091.zip › Slide09_tgaa091.tiff]

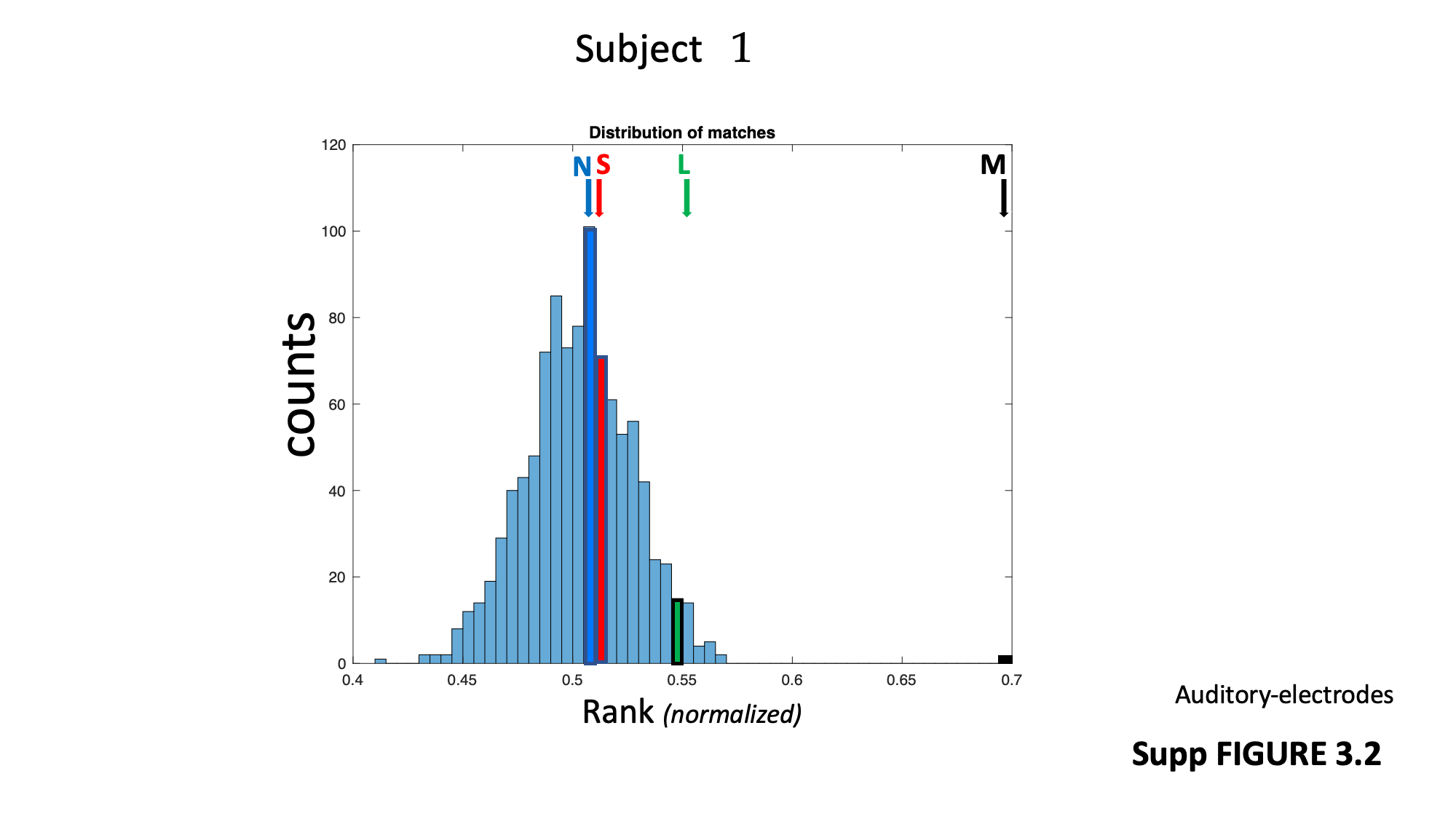

Supplement: Slide10_tgaa091 [file slide10_tgaa091.zip › Slide10_tgaa091.tiff]

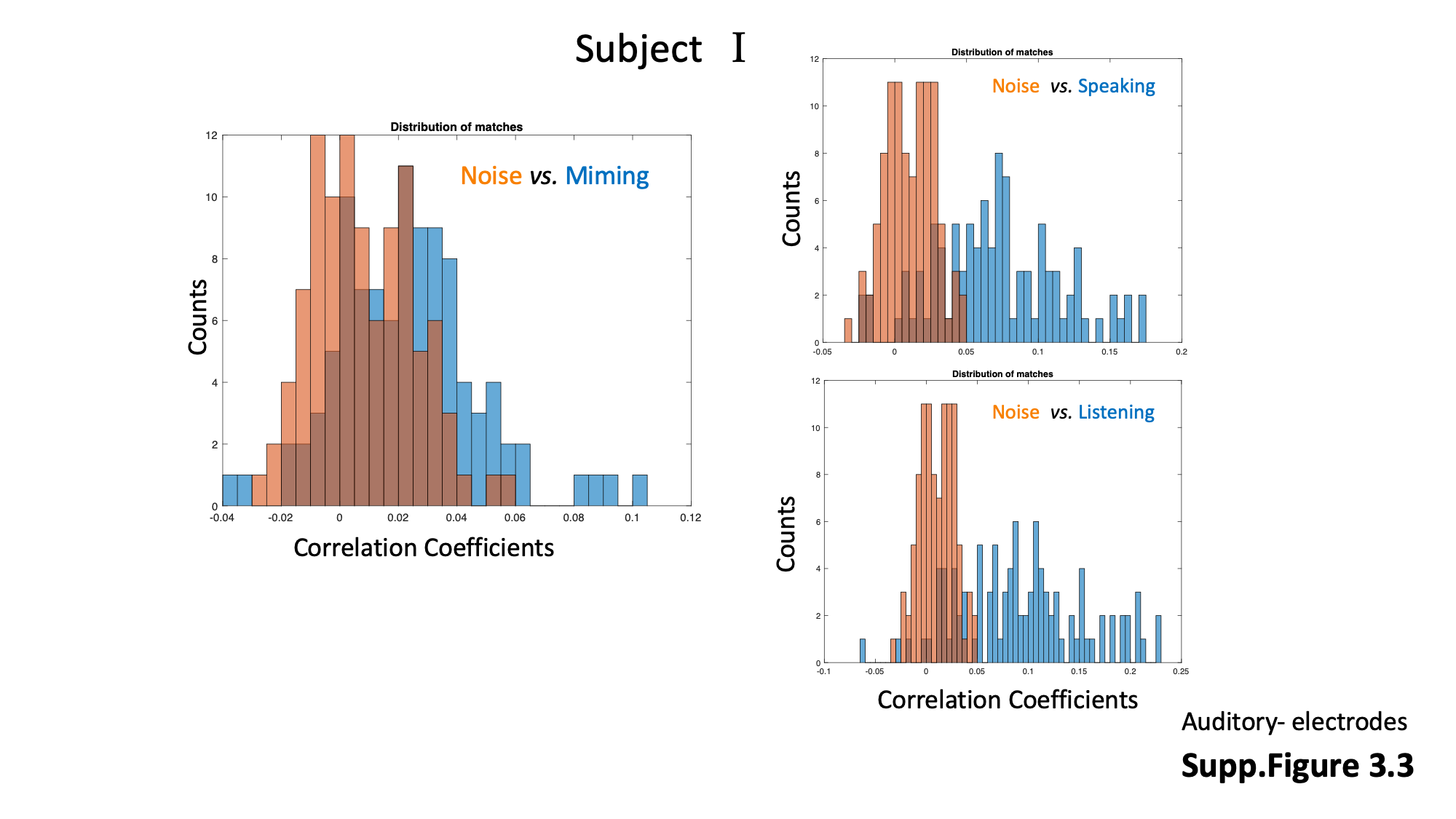

Supplement: Slide11_tgaa091 [file slide11_tgaa091.zip › Slide11_tgaa091.tiff]

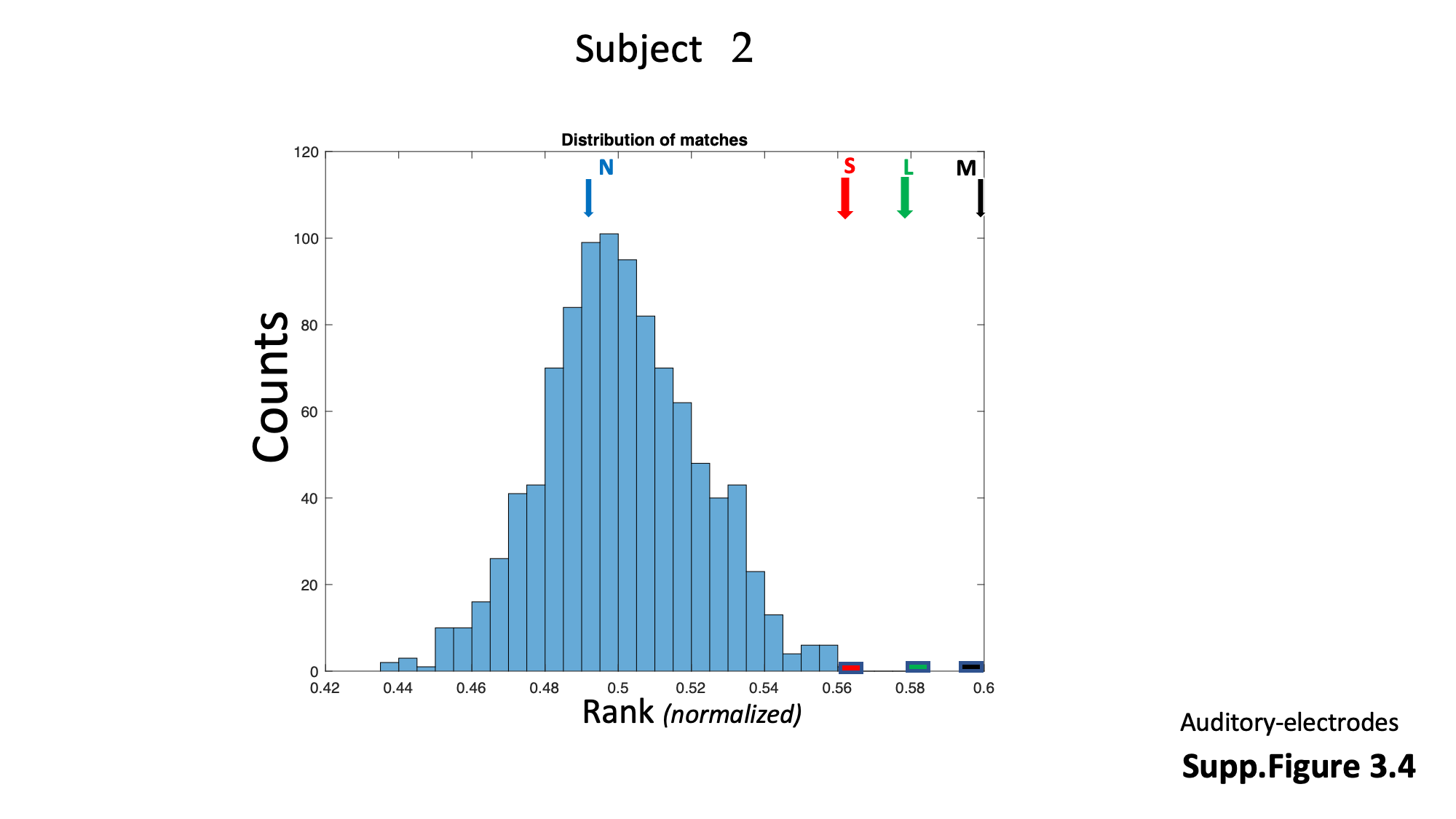

Supplement: Slide12_tgaa091 [file slide12_tgaa091.zip › Slide12_tgaa091.tiff]

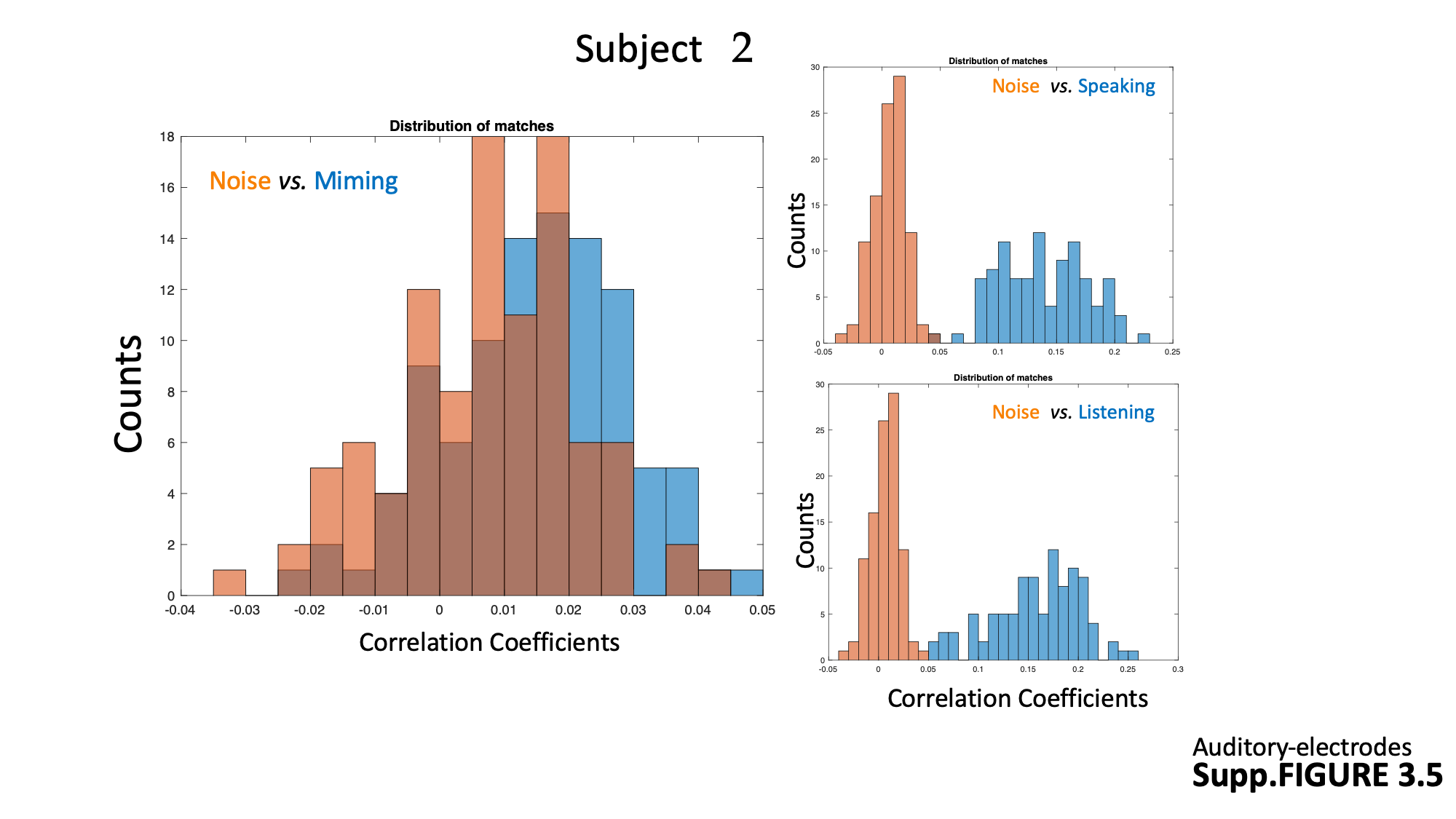

Supplement: Slide13_tgaa091 [file slide13_tgaa091.zip › Slide13_tgaa091.tiff]

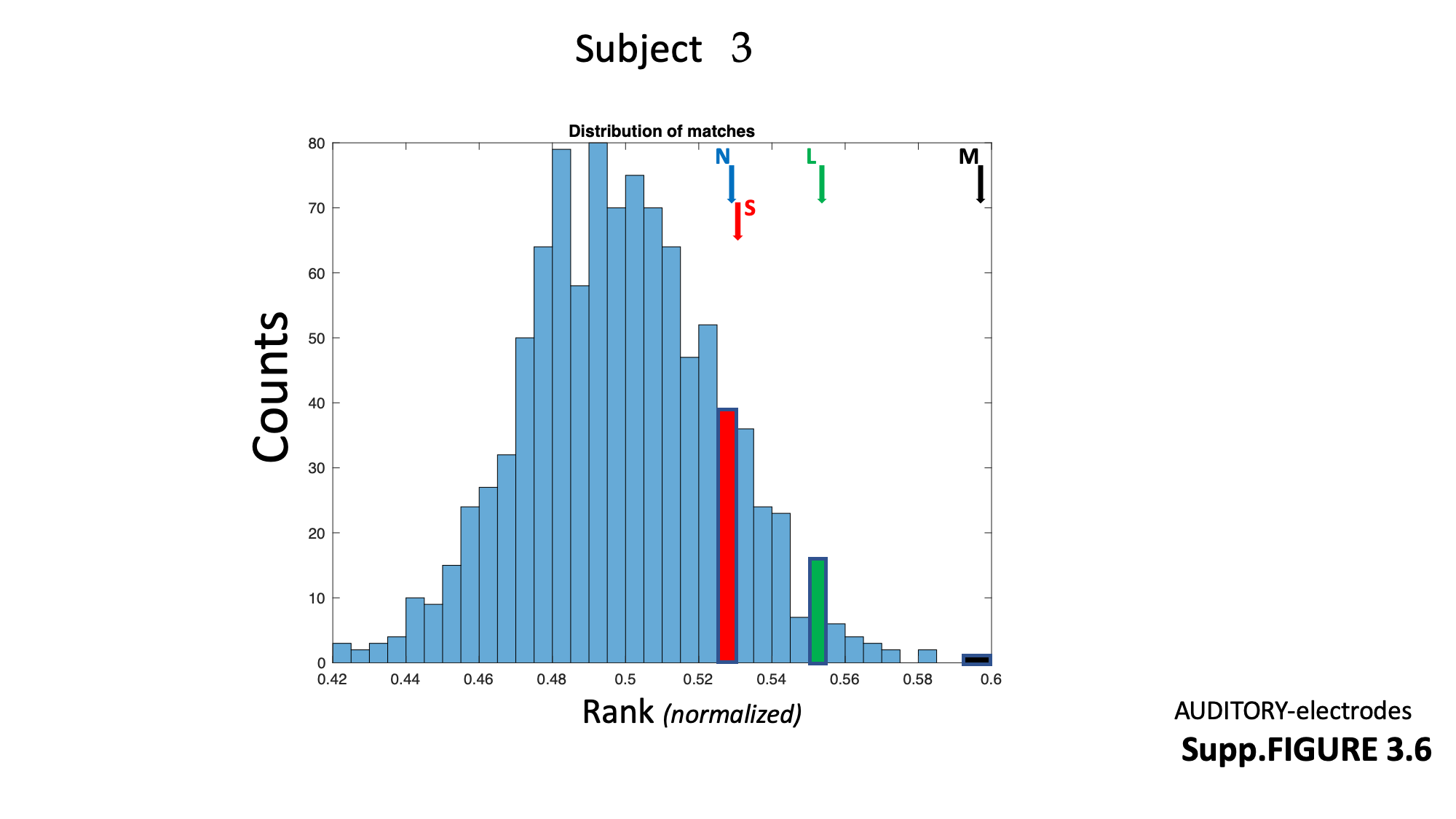

Supplement: Slide14_tgaa091 [file slide14_tgaa091.zip › Slide14_tgaa091.tiff]

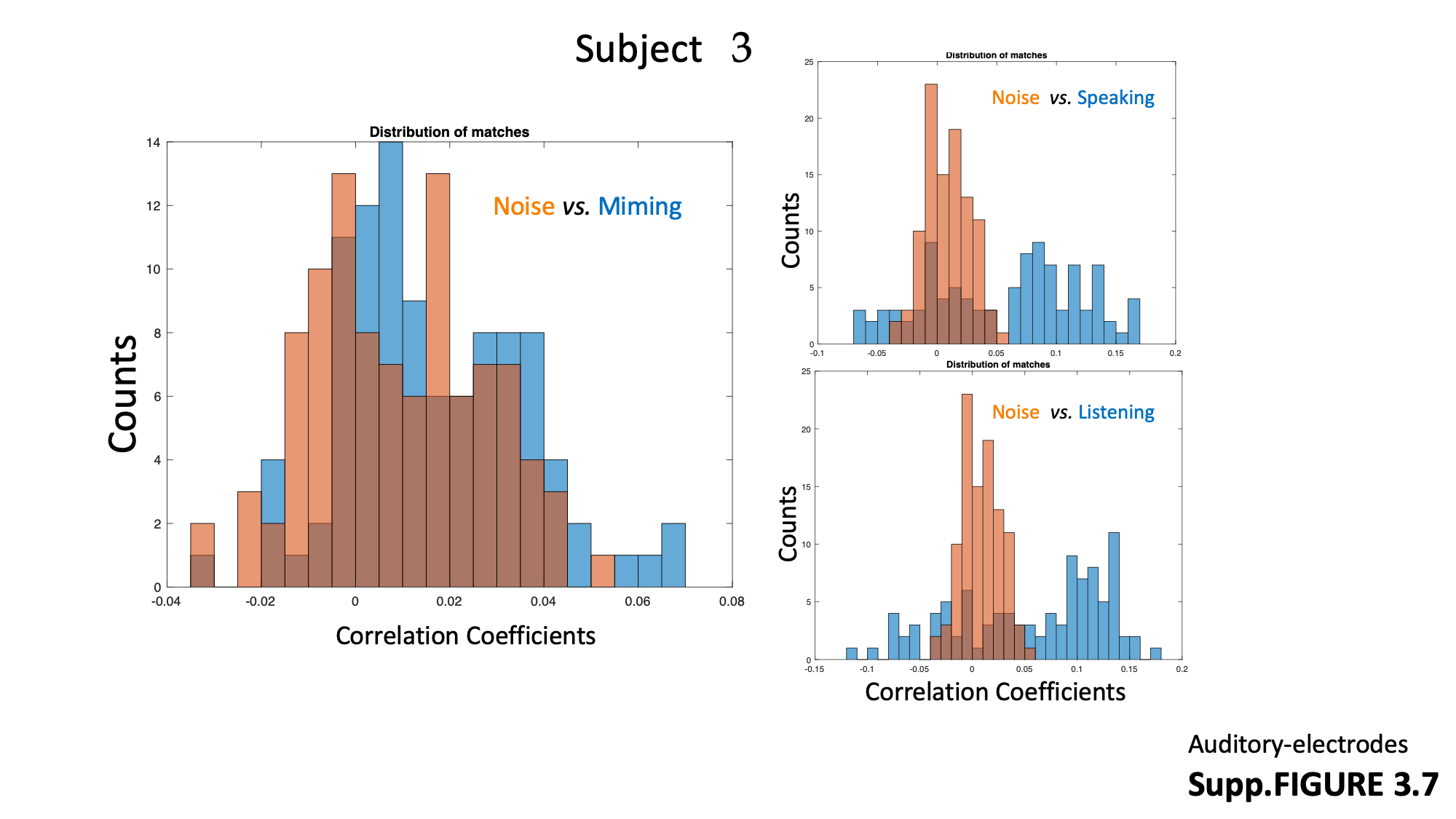

Supplement: Slide15_tgaa091 [file slide15_tgaa091.zip › Slide15_tgaa091.tiff]

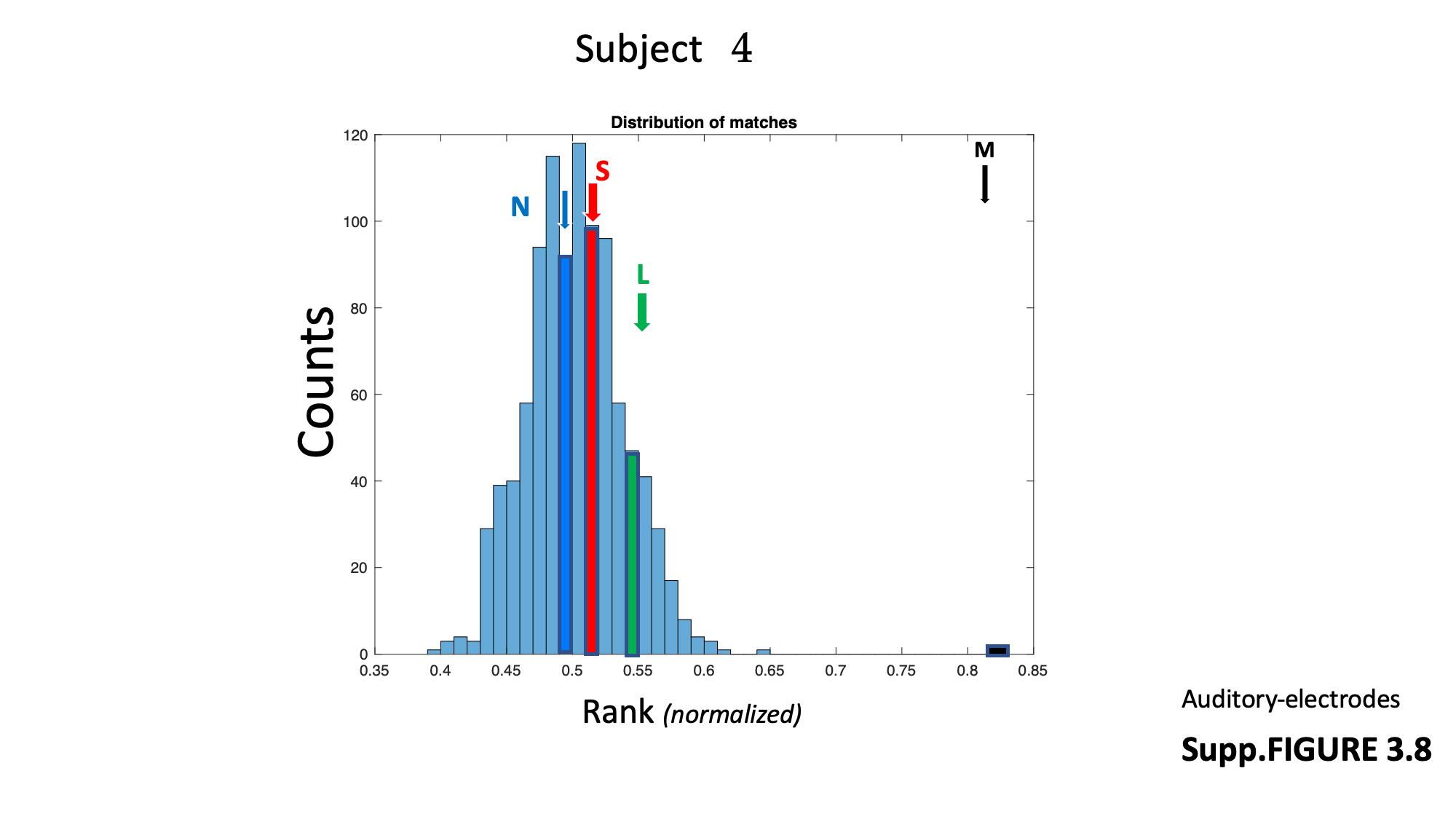

Supplement: Slide16_tgaa091 [file slide16_tgaa091.zip › Slide16_tgaa091.tiff]

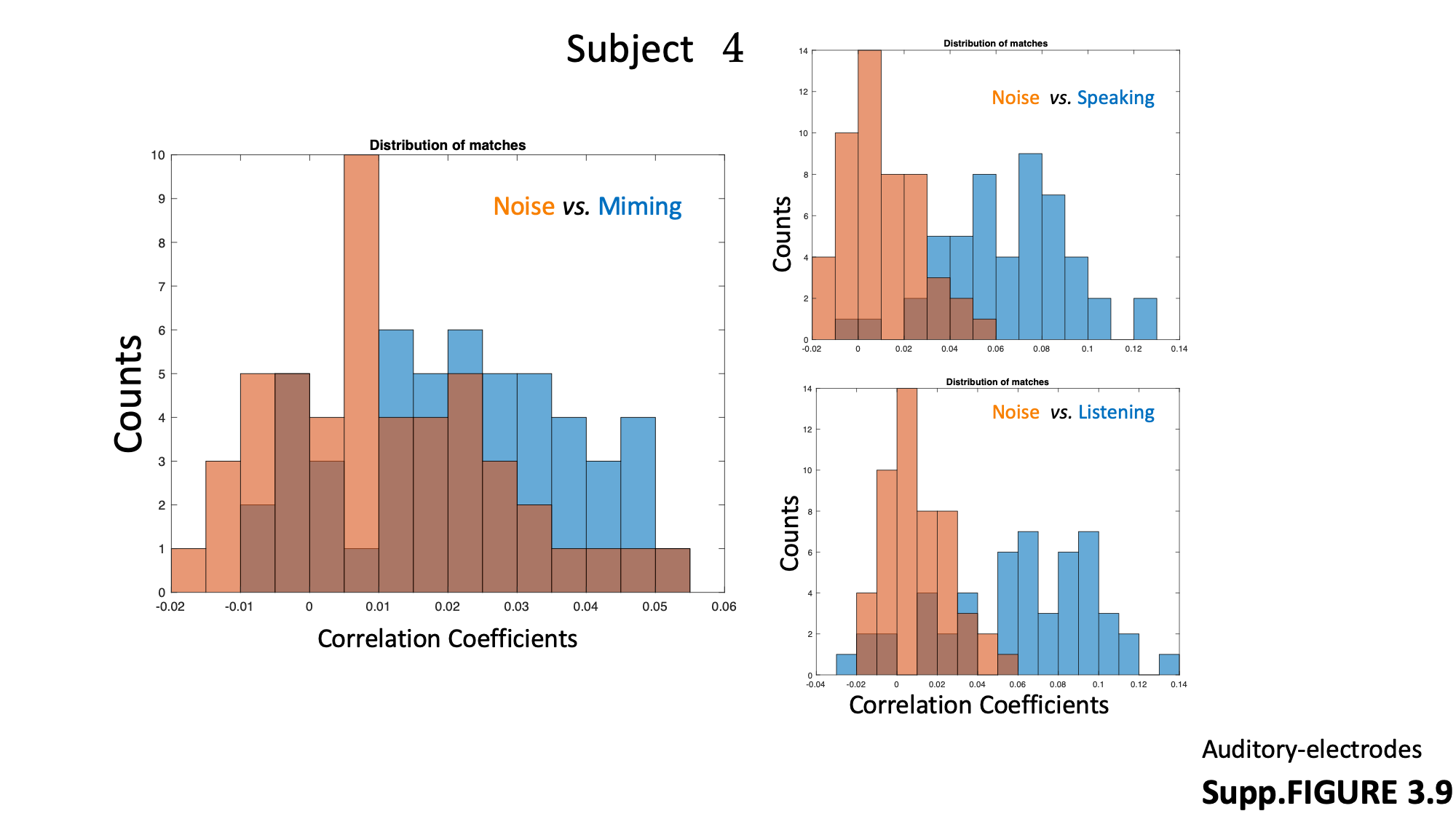

Supplement: Slide17_tgaa091 [file slide17_tgaa091.zip › Slide17_tgaa091.tiff]

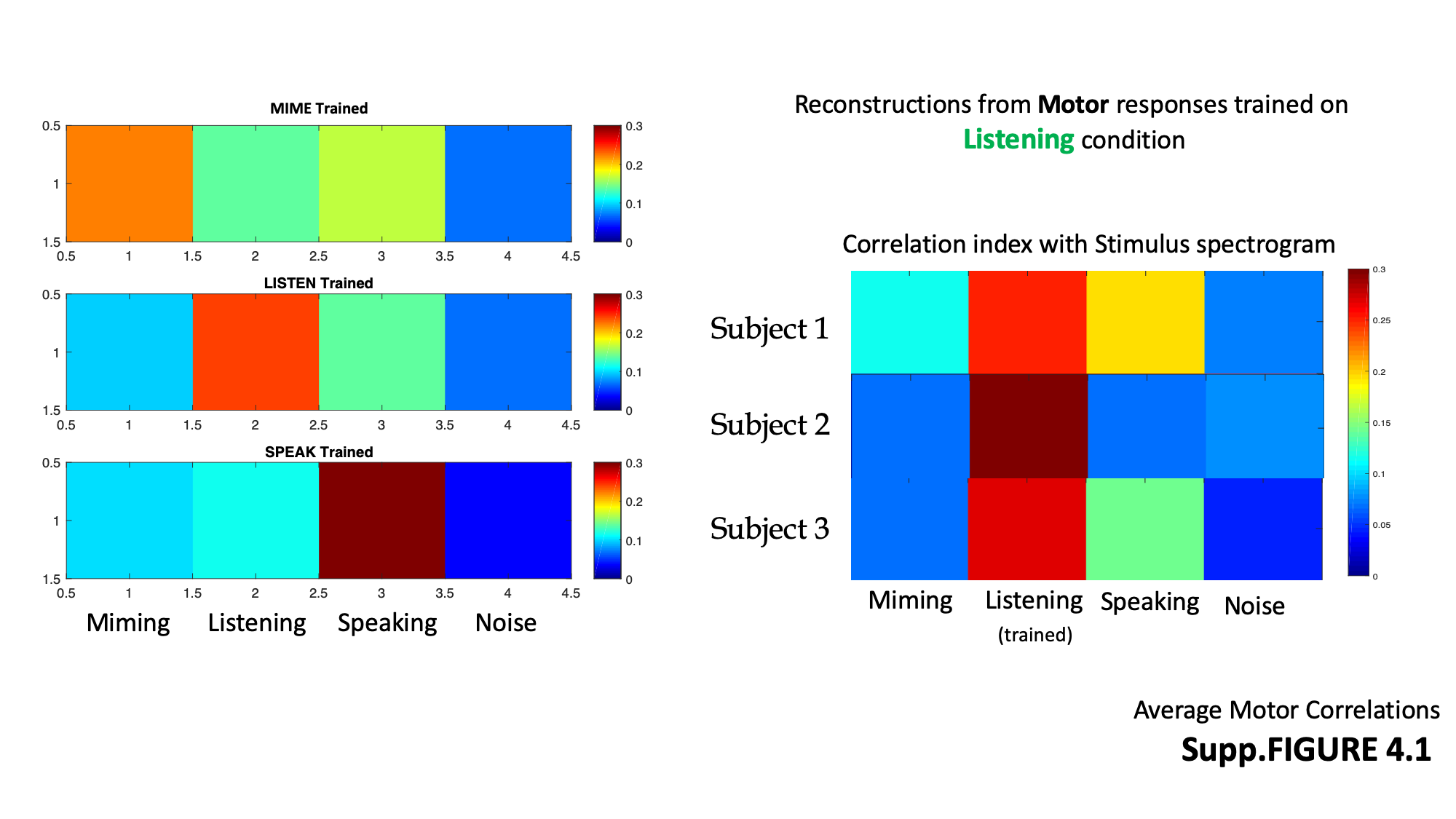

Supplement: Slide18_tgaa091 [file slide18_tgaa091.zip › Slide18_tgaa091.tiff]

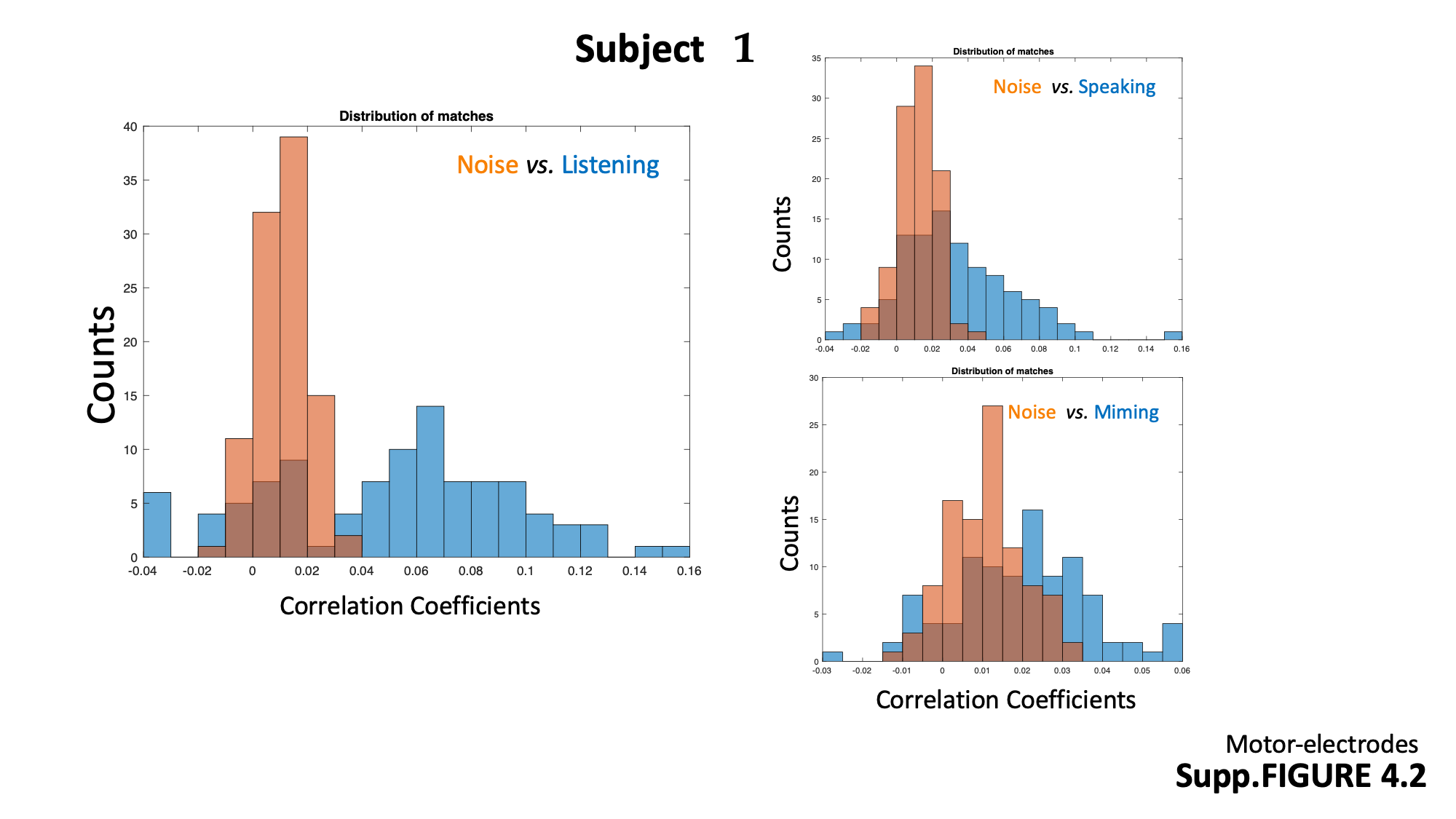

Supplement: Slide19_tgaa091 [file slide19_tgaa091.zip › Slide19_tgaa091.tiff]

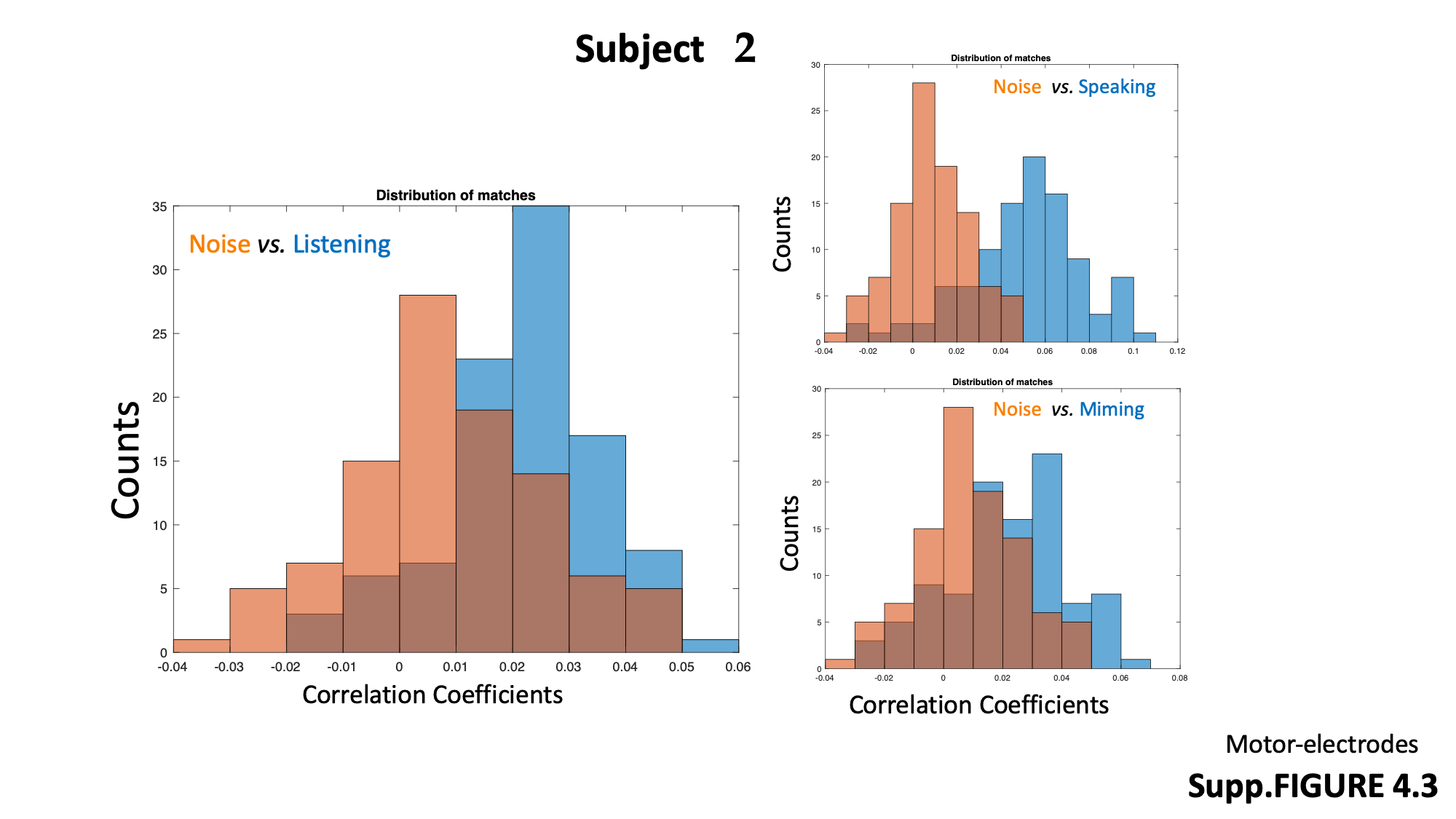

Supplement: Slide20_tgaa091 [file slide20_tgaa091.zip › Slide20_tgaa091.tiff]

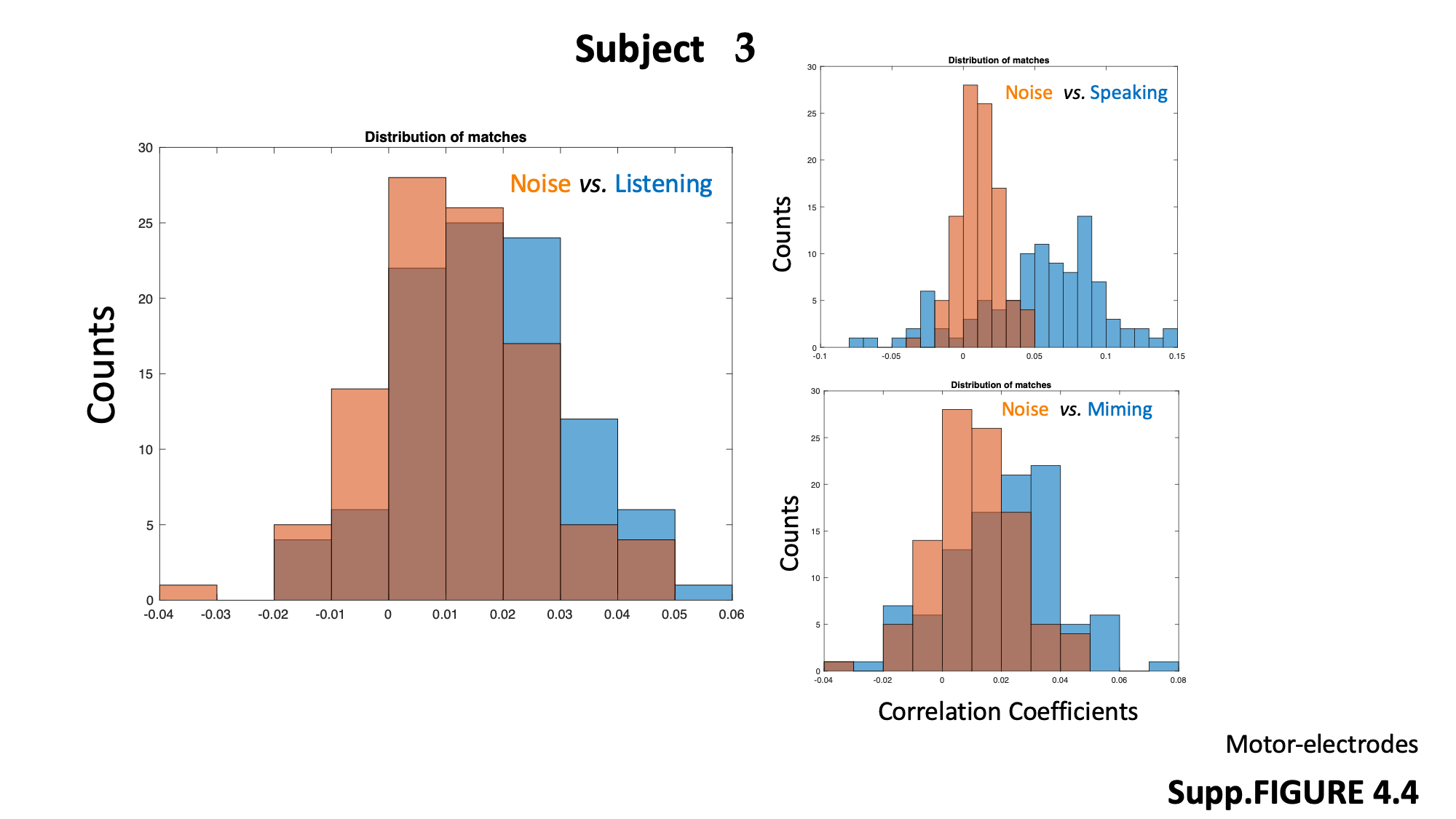

Supplement: Slide21_tgaa091 [file slide21_tgaa091.zip › Slide21_tgaa091.tiff]

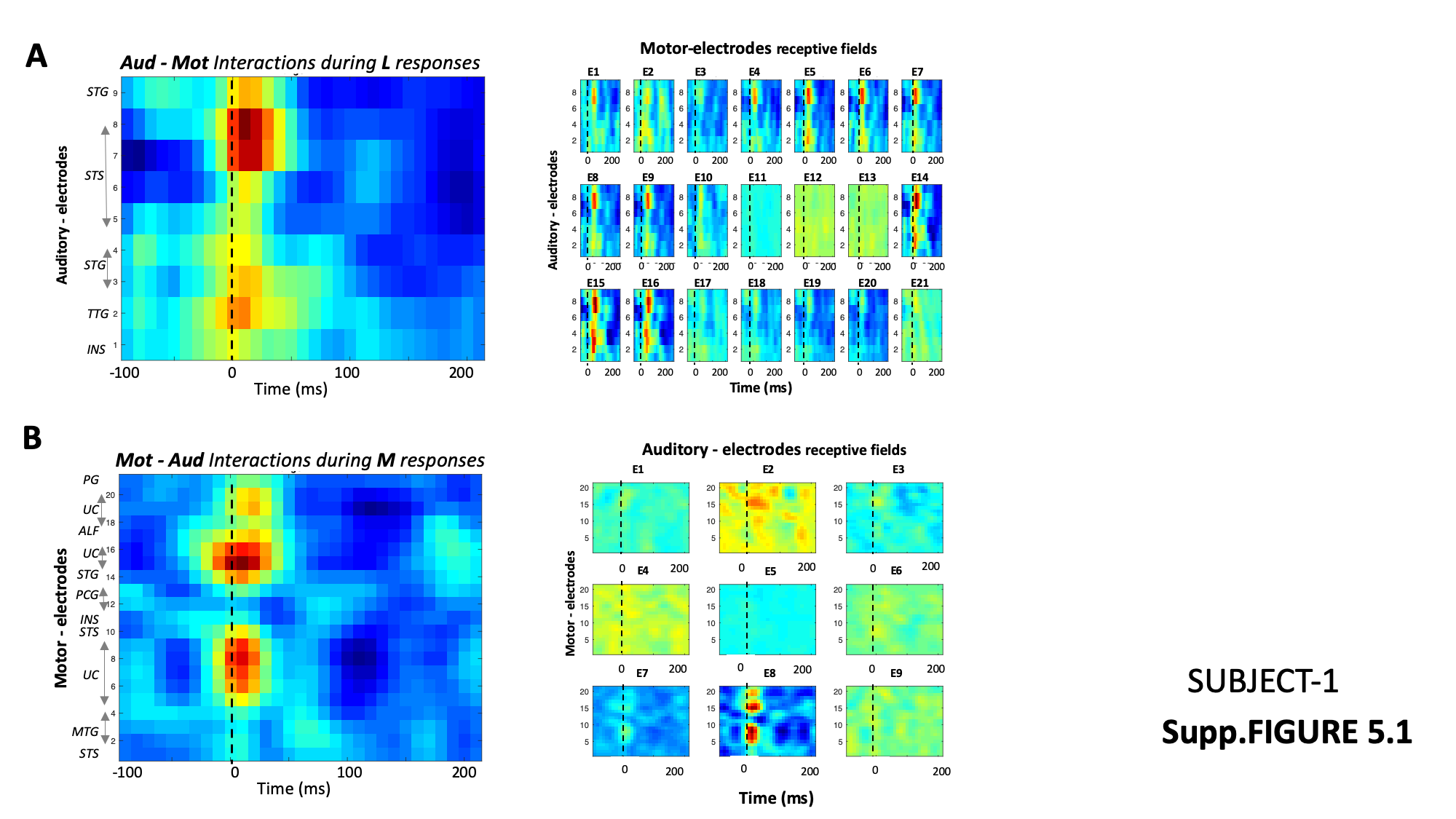

Supplement: Slide22_tgaa091 [file slide22_tgaa091.zip › Slide22_tgaa091.tiff]

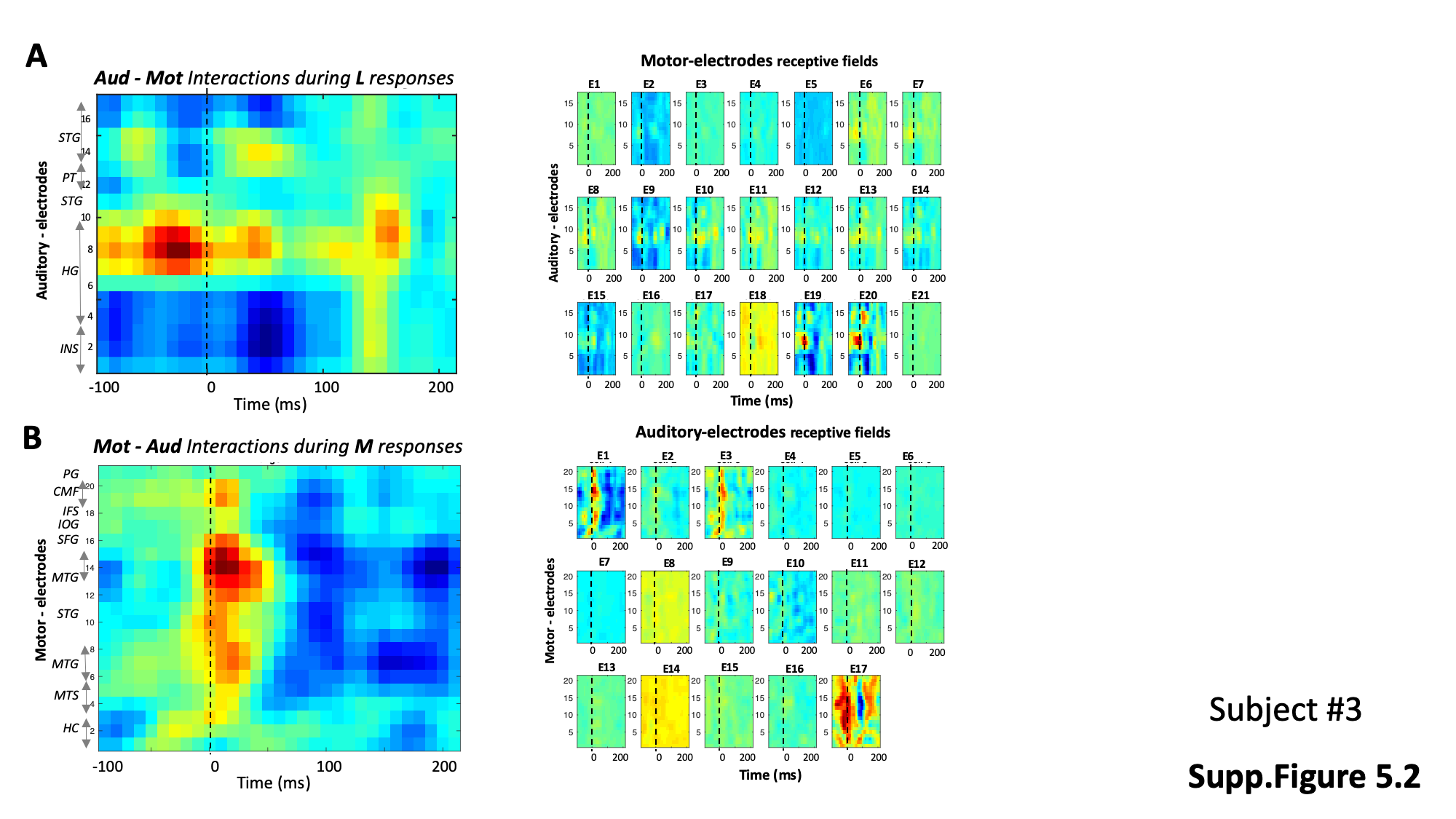

Supplement: Slide23_tgaa091 [file slide23_tgaa091.zip › Slide23_tgaa091.tiff]

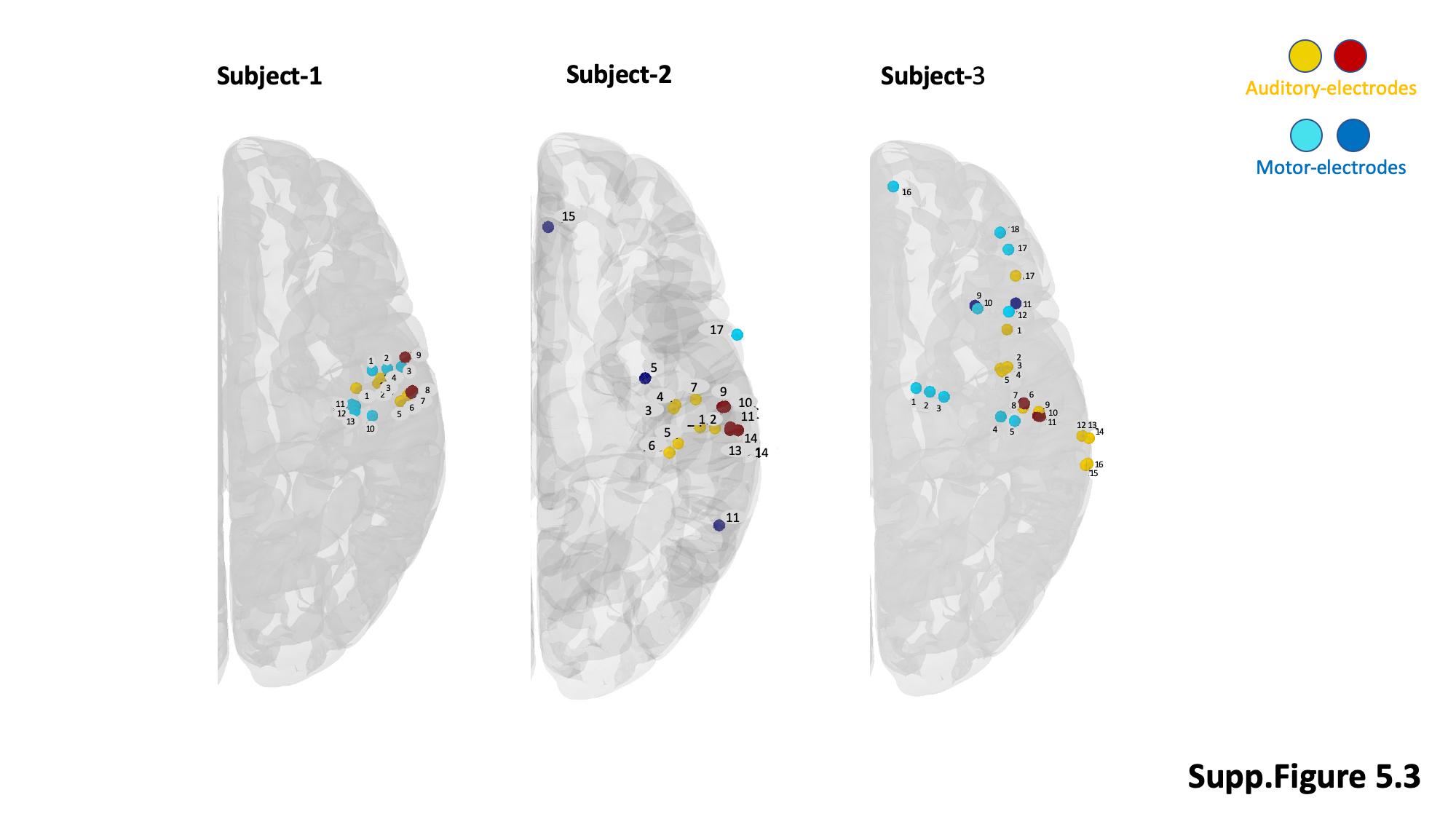

Supplement: Slide24_tgaa091 [file slide24_tgaa091.zip › Slide24_tgaa091.tiff]
